# Insights from draft genomes of *Heterodera* species isolated from field soil samples

**DOI:** 10.1186/s12864-025-11351-0

**Published:** 2025-02-18

**Authors:** Akshita Jain, Tongda Li, Daniel C. Huston, Jatinder Kaur, Conrad Trollip, John Wainer, Mike Hodda, Katherine Linsell, Ian T. Riley, Halil Toktay, Eniola Ajibola Olowu, Jacqueline Edwards, Brendan Rodoni, Timothy Sawbridge

**Affiliations:** 1 School of Applied Systems Biology, La Trobe University, Bundoora, VIC 3083 Australia; 2 Centre for AgriBioscience, Agriculture Victoria Research, Department of Energy, Environment and Climate Action (DEECA), Bundoora, VIC 3083 Australia; 3 Australian National Insect Collection, National Research Collection Australia, CSIRO, PO Box 1700, Canberra, ACT 2601 Australia; 4 Forest Science, NSW Department of Primary Industries, Parramatta, NSW 2150 Australia; 5 South Australian Research and Development Institute, Adelaide, SA 5064 Australia; 6 School of Agriculture, Food and Wine, The University of Adelaide, PMB 1, Glen Osmond, SA 5064 Australia; 7 Department of Plant Production and Technologies, Faculty of Agricultural Science and Technologies, Niğde Ömer Halisdemir University, Niğde, Turkey

**Keywords:** Cyst nematodes, Tylenchoidea, Heteroderidae, *Heterodera*, *Wolbachia*, Whole genome sequencing, Australian Biosecurity

## Abstract

**Background:**

The nematode phylum includes many species key to soil food webs with trophic behaviours extending from feeding on microbes to macrofauna and plant roots. Among these, the plant parasitic cyst nematodes retain their eggs in protective cysts prolonging their survival under harsh conditions. These nematodes, including those from the genus *Heterodera*, cause significant economic losses in agricultural systems. Understanding of nematode diversity and ecology has expanded through application of genomic research, however, for *Heterodera* species there are very few available whole genome sequences. Sequencing and assembling *Heterodera* genomes is challenging due to various technical limitations imposed by the biology of *Heterodera*. Overcoming these limitations is essential for comprehensive insights into *Heterodera* parasitic interactions with plants, population studies, and for Australian biosecurity implications.

**Results:**

We hereby present draft genomes of six species of which *Heterodera australis*,* H. humuli*, *H. mani* and *H. trifolii* are presently recorded in Australia and two species, *H. avenae* and *H. filipjevi*, currently absent from Australia. The draft genomes were sequenced from genomic DNA isolated from 50 cysts each using an Illumina NovaSeq short read sequencing platform. The data revealed disparity in sequencing yield between species. What was previously identified as *H. avenae* in Australia using morphological traits is now confirmed as *H. australis* and may have consequences for wheat breeding programs in Australia that are breeding for resistance to *H. avenae*. A multigene phylogeny placed the sequenced species into taxonomic phylogenetic perspective. Genomic comparisons within the *Avenae* species group revealed orthologous gene clusters within the species, emphasising the shared and unique features of the group. The data also revealed the presence of a *Wolbachia* species, a putative bacterial endosymbiont from *Heterodera humuli* short read sequencing data.

**Conclusion:**

Genomic research holds immense significance for agriculture, for understanding pest species diversity and the development of effective management strategies. This study provides insight into *Heterodera*, cyst nematode genomics and the associated symbionts and this work will serve as a baseline for further genomic analyses in this economically important nematode group.

**Supplementary Information:**

The online version contains supplementary material available at 10.1186/s12864-025-11351-0.

## Background

The roundworms, phylum Nematoda, represent a diverse and widespread group of organisms, inhabiting all ecosystems including soil, marine and freshwater environments [1]. With over 25,000 described species, they play an essential role in nutrient cycling and soil ecology. Of these, 4,100 species are described as plant parasitic nematodes (PPNs), causing significant economic losses in agricultural systems through infection of host roots [2]. Notable PPNs include the root-knot nematodes (*Meloidogyne* spp.), cyst nematodes (*Globodera* and *Heterodera* spp.) and root-lesion nematodes (*Pratylenchus* spp.) [3].

Cyst nematodes of the family Heteroderidae are considered the second most damaging group of nematode pests globally after the root-knot nematodes [3]. Their unique life cycle involves the formation of tough protective cysts through the process of polyphenol oxidase tanning around the female’s body after its death [4]. Cysts, containing hundreds of embryonated eggs, enable them to withstand adverse environmental conditions and prolong their survival. Within these cysts, eggs hatch into infective J2 juveniles, initiating the parasitic phase of their life cycle where they locate and penetrate host plant roots, eventually establishing a feeding site that facilitates nutrient uptake for the juvenile [5]. Cyst nematodes exhibit a wide host range, affecting an array of crops including cereals, legumes and vegetables, but individual species are relatively host specific [5, 6]. The impact of cyst nematodes on ecosystems extends beyond direct agricultural effects, since these parasites can influence soil health, nutrient cycling and microbial communities and composition [7]. *Heterodera* Schmidt, 1871, is the largest genus in the Heteroderidae and consists of at least 80 recognised species [8]. Historically, *Heterodera* species were categorised into three species groups based on their vulval cone structures, namely, *Avenae*, *Goettingiana* and *Schachtii* [9]. Subbotin, Vierstraete et al. [10] considered a combination of molecular data along with the morphological characteristics of the vulval cones to support the recognition of four species groups with some modifications to the species composition, *Avenae*, *Goettingiana*, *Humuli* and *Schachtii*, and introduced two new groups, *Cyperi* and *Sacchari*. Following the merging of the *Afenestrata* genus with *Heterodera*, the addition of the *Afenestrata* species group within *Heterodera* was also proposed by Mundo-Ocampo, Troccoli et al. [11]. Both morphological and molecular analyses further affirm this division of *Heterodera* into seven distinct species groups [9].

Most early reports of *Heterodera* infestations were primarily from Europe and North America and causing significant damage to cereal crops [12]. In recent decades there has been a global expansion in the distribution of *Heterodera* parasites affecting many economically important crop types and raising concerns within agricultural communities [12]. In Australia, species of *Heterodera* are known to affect a diverse range of crops including wheat, barley, hop, cabbage and cauliflower, posing a considerable threat to Australia’s agricultural sector [9]. One pathotype of one species of *Globodera*,* G. rostochiensis* (Ro1), and nine *Heterodera* species, namely, *H. australis*,* H. cruciferae*,* H. daverti*,* H. fici*,* H. graminis*,* H. humuli*,* H. mani*,* H. schachtii* and *H. trifolii*, are currently recorded in Australia [12–16].

According to Beale, Fairbrother et al. [17], biosecurity is defined as the “protection of the economy, environment and human health from the negative impacts associated with entry, establishment or spread of exotic pests (including weeds) and diseases”. The main objective of implementing stringent biosecurity practices is to guard against the threats that diseases and organisms represent. Until recently, PPNs were not given enough attention as biosecurity hazards. This is partially due to the fact that PPNs are minute, often live in the soil and are rather reductive than destructive to crop yield, and are challenging to research and monitor [18]. However, biosecurity measures are crucial in order to stop PPNs from entering and spreading throughout agricultural fields, which might endanger global food security by lowering crop yields and promoting the spread of disease [19]. The National Priority Plant Pests (NPPP) list (2019) [20] of Australia is a compilation of the top forty plant pests that are regarded as the most significant threats to the country’s agricultural sector. Developed collaboratively by the Australian federal, state and territory governments, along with industry stakeholders, the NPPP list serves as a strategic tool for prioritising resources and efforts in plant biosecurity. Potato cyst nematode (*Globodera* spp.) and cyst nematodes of grains and vegetables (*Heterodera* spp. including *H. carotae*,* H. filipjevi*, *H. glycines*, *H. latipons*, *H. sorghi*, *H. zeae*) [21] are included on the NPPP list and are therefore a focus for national preparedness capability through the development of national action plans.

Genomics has substantially contributed to the understanding of nematode biology and their interactions with other organisms [22]. Whole genome sequencing (WGS) is a powerful and comprehensive technique that involves determining the complete DNA sequence of a genome. It offers a thorough view of their genetic makeup, revealing genes that may be associated with parasitism, virulence and host interactions [22]. Since 2011, the advent of next generation sequencing has facilitated the comprehensive genome sequencing of numerous economically important PPNs, including the pinewood nematode *Bursaphelenchus xylophilus* [23], potato cyst nematodes *Globodera pallida* [24] and *G. rostochiensis* [25], *G. ellingtonae* [26], potato tuber nematode *Ditylenchus destructor* [27], stem and bulb nematode *Ditylenchus dipsaci* [28], banana root nematode *Pratylenchus coffeae* [29], *Pratylenchus scribneri* [30], *Rotylenchulus reniformis* [31], *Radopholus similis* [32] and several species of *Meloidogyne* [33–38]. As technology continues to evolve, ongoing genomic research promises to provide even more insights into the complex world of nematodes [39].

In 2019, the first draft genome of a species of *Heterodera* was published. The genome of the soybean cyst nematode *Heterodera glycines* was estimated to be 123,846,405 base pairs (bp), containing 738 contigs with an N50 of 304 Kbp [40]. In 2021, a more complete assembly of *H. glycines* was published with a revised genome size of 157,982,452 bp consisting of nine scaffolds and 2,109 contigs with an N50 of 17.9 Mbp (17,908,190 bp) [41]. A year later, the genome of the sugar beet cyst nematode *H. schachtii*, was sequenced and published [42]. This is the largest cyst nematode genome published to date with a final assembly length of 179 Mbp consisting of 395 scaffolds [42]. In 2022, the genome of the carrot cyst nematode *H. carotae* was sequenced and published from a biological input of 50 cysts, with an assembly size of 95.1 Mbp contained within 17,839 scaffolds, annotated for 17,212 protein coding genes [43]. More recently, in July 2024, a high-quality chromosome-level genome assembly for *H. filipjevi* using Illumina, PacBio and Hi-C sequencing was produced. The assembled genome comprised of nine pseudo-chromosomes spanning over 134.19 Mb with a scaffold N50 of 11.88 Mb, annotated for 10,036 protein coding genes [44]. Although *Heterodera* species play a significant role in agricultural systems, there is a noticeable scarcity of whole genome data in comparison with other PPNs such as the root-knot nematodes [33, 45].

Sequencing the genomes of *Heterodera* species presents challenges due to their complex genomes, characterized by repetitive DNA sequences, and their distinctive biology. While the inaccessibility of certain life stages is less critical for genome sequencing itself, it poses a significant obstacle when aiming to complement genomic data with a comprehensive transcriptome. The parasitic stages of *Heterodera* species, which occur inside the host plant, make it difficult to obtain sufficient material for transcriptome analysis, thereby limiting insights into gene expression across different developmental stages [46]. The isolation of high molecular weight DNA is a crucial step for successful WGS [47]. Furthermore, the cysts are exceptionally small and can survive in soil for more than 20 years, presenting a challenge as the DNA quantity and quality can vary due to the unknown age of the collected samples [48]. In light of this limitation, researchers have resorted to pooling cysts and J2 juveniles for sequencing, a practice that could potentially result in the loss of haplotypic information and impede the identification of genetic markers [22, 49]. Technical constraints such as bioinformatic algorithms utilised in genome assembly pipelines are challenging due to the low GC content prevalent in the nematode genomes. This hinders precise differentiation between authentic genomic variations and sequencing errors, ultimately affecting the accuracy of the assembled genome [46, 50, 51], further complicating genome annotation and the interpretation of sequencing results. Nevertheless, WGS is particularly important for understanding plant-parasitic interactions, while also offering valuable insights into the genetic diversity and structure of populations. The objective of this study was to sequence, assemble and assess draft genomes of six *Heterodera* species from an input of 50 cysts per species, encompassing four species recorded in Australia and two species of biosecurity significance not known to occur in Australia. This genome sequence dataset will provide the nematology community with a repository of *Heterodera* genomes to facilitate progress in comparative and functional genomics within this group. The genomic data hence generated could further be used to determine diagnostic research and capability building to manage *Heterodera* species of Australian biosecurity concern.

## Methods

### Cyst material sampling

Cysts of six *Heterodera* species– *H. australis*,* H. avenae*,* H. filipjevi*,* H. humuli*,* H. mani* and *H. trifolii* were acquired as source biological material for genomic DNA extraction and library preparation for short read sequencing (Table 1). Ethanol preserved cysts are allowed to be imported under the Australian biosecurity protocols for exotic species and therefore provided a route to study these exotic cyst nematode genomes within Australia. All acquired cysts (exotic cysts were imported under DAFF import permit: 0007604708) were extracted from soil samples [52] and were stored in 100% ethanol in Eppendorf tubes at 4 °C.

**Table 1:** Origin, life stage, voucher accession, source and DNA concentration of *Heterodera* species sequenced in this study

S. no.	VPRI^1^ Accession	Species^2^	Common name	Species group	Location	Life stage sequenced	DNA Concentration (ng/µl)	Source
1	44,576	*Heterodera australis*	Australian cereal cyst nematode	*Avenae*	South Australia, Australia	Cysts, second-stage juveniles	2.2	K Linsell
2	44,577	*Heterodera avenae* ^3^	Cereal cyst nematode	*Avenae*	Hatay Province, Turkey	Cysts, second-stage juveniles	0.79	IT Riley, H Toktay, EA Olowu
3	44,578	*Heterodera filipjevi* ^4^	Filipjev’s cereal cyst nematode	*Avenae*	Nevesehir Province, Turkey	Cysts, second-stage juveniles	0.6	IT Riley, H Toktay, EA Olowu
4	44,579	*Heterodera humuli*	Hop cyst nematode	*Humuli*	Tasmania, Australia	Cysts, second-stage juveniles	0.703	DC Huston, M Hodda
5	44,580	*Heterodera mani*	Ryegrass cyst nematode	*Avenae*	Tasmania, Australia	Cysts, second-stage juveniles	lower than blank^5^	A Jain, J Wainer
6	44,581	*Heterodera trifolii*	Clover cyst nematode	*Schachtii*	Canberra, Australia	Cysts, second-stage juveniles	0.185	DC Huston, M Hodda

^1^Victorian Plant Pathogen Herbarium (VPRI) accession number

^2^Molecular identification for all species confirmed using the cytochrome oxidase I (COI) gene region marker

^3^Exotic species for Australia, according to Huston, Khudhir et al. [21]

^4^Exotic species for Australia listed on the National Priority Plant Pests (NPPP) list endorsed by Australian Government, Department of Agriculture, Fisheries and Forestry [20]

^5^Lower than blank indicates that the DNA concentration of *H. mani* was lower than the detection limit of Quantus Fluorometer

### Preparation and molecular identification of genomic DNA

Genomic DNA from a pool of 50 cysts from each of the six *Heterodera species* (Table 1) was extracted using the QIAamp Micro DNA Extraction kit (Qiagen, Hilden, Germany) following the manufacturer’s protocol with one modification where the cysts were mixed with the extraction buffer and proteinase K in a Thermomixer^®^ (Eppendorf©, Hamburg, Germany) overnight at 800 rpm at 56 °C. DNA was eluted in 30 µl elution buffer and quantified using Quantus Fluorometer (Promega, Madison, WI, USA). Molecular identification of the cysts was performed using the COI gene region using the protocol detailed in Jain, Wainer et al. [15].

### Illumina library construction and sequencing

Libraries with an average insert size of 300 bp were generated using the NEBNext FS Ultra II DNA kit (New England BioLabs^®^, Ipswich, MA, USA) without size selection and the following modifications. During the adaptor ligation step, the adaptors were diluted to 1:25 ratio as described for the low input library preparation and a qPCR was performed instead of a PCR to add adaptors and avoid over amplification. DNA quantity and length was determined using High Sensitivity D1000 ScreenTape (Agilent Technologies, Santa Clara, CA, USA) on 4200 TapeStation. All samples were sequenced using Illumina NovaSeq 6000 (Illumina, San Diego, CA, USA) according to the manufacturer’s instructions [53]. Five of the six libraries were sequenced with the NovaSeq S1 Flow Cell (2 × 150 bp), whereas the *H. filipjevi* genomic DNA library was sequenced using the NovaSeq SP Flow Cell (2 × 250 bp). All libraries were treated with the Illumina Free Adapter Blocking Reagent to reduce sequencing errors due to index switching.

### Assembly and quantification

Raw sequencing reads were quality checked and trimmed using fastp using default settings [54]. Using the raw read as input and a kmer size of 21, GenomeScope [55] was used to determine the potential genome size and repeat length for the six sequenced species. Following quality trimming, initial de novo genome assemblies were produced using SPAdes v3.15.5 [56]. Assemblies were performed using error corrected reads with a kmer range of 21, 33, 55, and 77. The raw genome assemblies were decontaminated, firstly by using Redundans [57] to remove additional haplotypes present in the assembly, followed by BlobTools [58] to identify and remove bacterial and other contaminants present in the assemblies using the NCBI BLAST TAXID database [59]. Only scaffolds containing hits for “Nematoda” or “no-hit” (sequences with unknown origin, however, which could potentially be novel nematode sequences) were retained. The no-hit reads and scaffolds were further analysed using a Kraken2 [60] pipeline to filter out any potential bacterial and fungal contaminants (Supplementary Table 1) using custom built databases containing NCBI RefSeq bacterial and fungal genomes with the following commands. For reads: --db RefSeq 22,022,022 --threads 16 --use-names --output sample.kraken --report sample.kraken.report --paired --gzip-compressed R1.fastq.gz R2.fastq.gz, for assembly: kraken2 --db RefSeq 22,022,022 --threads 16 --use-names --output sample.kraken --report sample.kraken.report --unclassified-out unmapped_contigs.fasta assembly.fasta. These filtered bacterial and fungal contigs were examined in the context of the metagenomic analysis associated with the cysts.

**Fig. 1:**
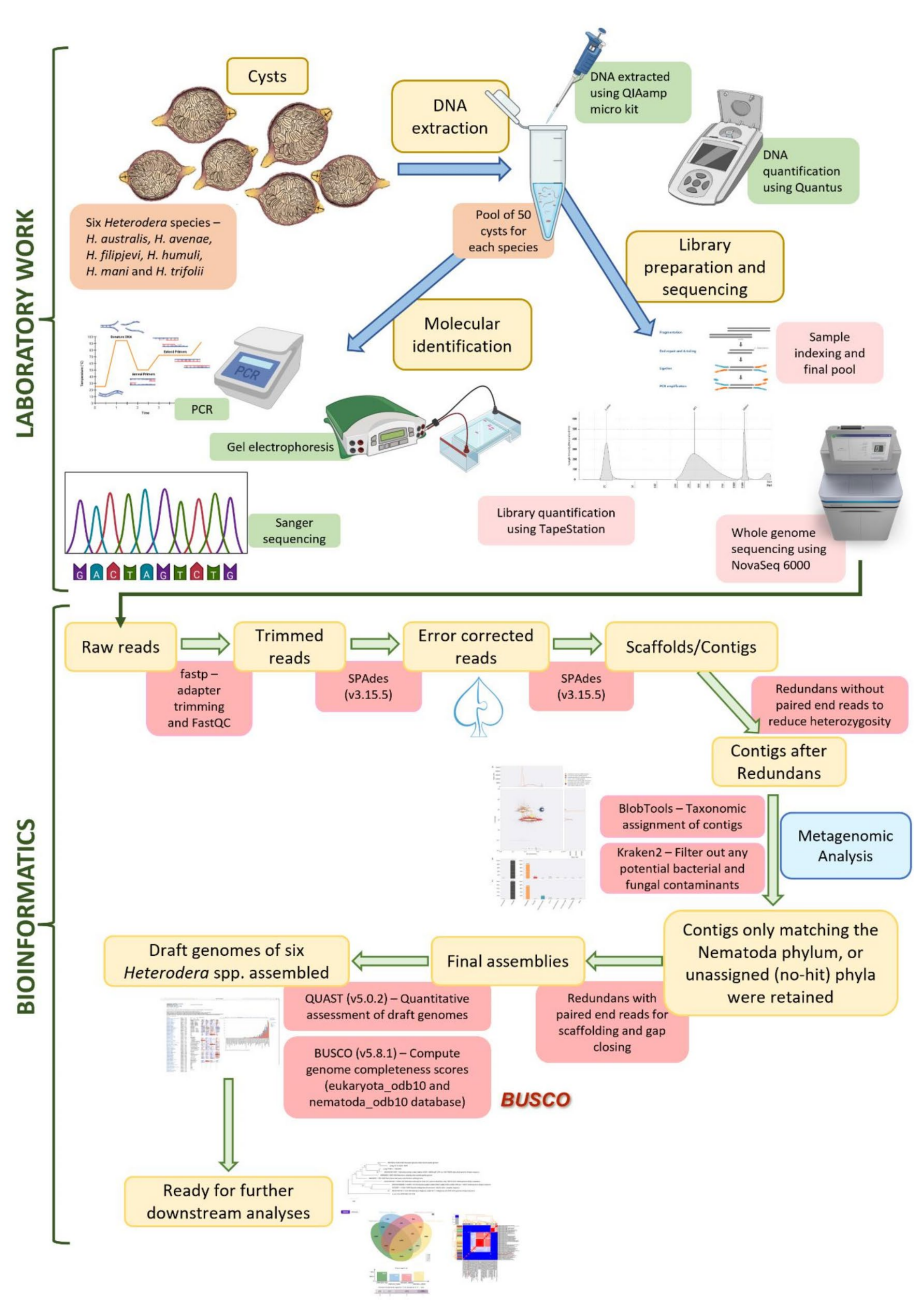
Graphical representation of the draft genome assembly pipeline– from field sample and wet-lab procedures to bioinformatics. Cysts were drawn using Apple Procreate^®^, other figures were created using BioRender (paid licence) [63]

A second round of Redundans [57] on the Nematoda-related scaffolds was conducted to fill gaps using the paired end reads and contig segments below 200 bp were removed using seqtk [20]. The final draft genome assemblies were subjected to genome quality assessments using QUAST v5.0.2 [61]. Genome completeness assessments were performed using BUSCO v5.8.1 [62] using both eukaryotic and lineage specific (Nematoda) databases. A graphical representation of the draft genome assembly pipeline is presented in Fig. 1.

### Annotation and phylogenetic analysis of *Heterodera* species

Assembled draft genomes of the *Heterodera* species were annotated using Augustus v3.4.0 [64] using *Heterodera schachtii* as the model species. The model was trained using the annotations and complete genome of *Heterodera schachtii* available on the WormBase Parasite website [65] using the following script autoAugTrain.pl --genome = heterodera_schachtii.PRJNA522950.WBPS19.genomic.fa --trainingset = heterodera_schachtii.PRJNA522950.WBPS19.annotations.gff3 --species = hschachtii --cpus = 8.

Using the BLAST+ (Basic Local Alignment Search Tool) module [66], a BLAST database was created with the local sequences obtained from the sequenced *Heterodera* species using makeblastdb -in dbname.fa -dbtype nucl -parse_seqids [67] to extract three molecular markers of interest: the heat shock protein 90 (*hsp*90) gene region, the mitochondrial cytochrome c oxidase I (mt COI) gene region, and the large subunit ribosomal RNA (28 S rRNA). The sequences were extracted using published accession numbers available on NCBI (National Center for Biotechnology Information) GenBank through a nucleotide blast of the query sequences against the local BLAST database created earlier using blastn -query seq.fa -db dbname.fa. Alignments of *hsp*90, mt COI and 28 S rRNA gene sequences were conducted using MAFFT v7.450 [68, 69] with default parameters in Geneious Prime [70]. The extracted sequences (Supplementary Table 2) from each gene region for each of the *Heterodera* species were aligned with selected sequences from NCBI GenBank for *hsp*90, mt COI and 28 S rRNA gene regions. Majority-rule consensus trees were constructed through Bayesian Inference (BI) and maximum likelihood (ML) analyses. Bayesian inference was conducted using MrBayes v3.2.6 [71] with default parameters using the GTR Gamma I nucleotide substitution model. Maximum likelihood analyses were performed using RAxML v8 [72] with 1000 bootstraps replicates along with rapid bootstrapping and search for the best-scoring ML tree algorithms. Combined BI/ML trees were edited using Microsoft PowerPoint.

Orthologous gene clusters for *Heterodera* species belonging to the *Avenae* species group were identified and analysed through OrthoVenn3 [73] using the OrthoMCL algorithm. The annotated protein sequences from the *Avenae* species group– *H. australis*,* H. avenae. H. filipjevi* and *H. mani* were taken as the input and processed using default settings with an E value of 1e-2 and the inflation value of 1.5 to balance sensitivity and selectivity of the clusters.

### *Wolbachia* draft genome assembly and analysis from *Heterodera Humuli* metagenomic sequence data

*Wolbachia* reads from the *H. humuli* metagenomic dataset were extracted using BBMap [74] with a custom perl script, employing a closely related *Wolbachia* genome (isolate: *w*Tex, GenBank Accession number GCA_022836975.1) [75] downloaded from NCBI GenBank. SPAdes v3.15.5 [56] was then utilised to assemble the reads, and QUAST v5.0.2 [61] quantified the final draft genome assembly. ANI [76] analysis was conducted using 31 different *Wolbachia* isolate whole genome sequences (Supplementary Table 3) downloaded from NCBI GenBank along with the newly generated draft genome of *Wolbachia* (isolate: *w*Hhum) using the pyANI [77] Conda environment.

## Results and discussion

*Heterodera* species are endoparasitic, sedentary, biotrophic pathogens having intimate interactions with their plant hosts [9]. Genomic and phylogenomic studies are critical tools to aid the development of pest management strategies since *Heterodera* spp. remain formidable agriculture pests globally causing substantial crop losses [3]. The assembly and annotation of draft genomes for six *Heterodera* species– *H. australis*,* H. avenae*,* H. filipjevi*,* H. humuli*,* H. mani* and *H. trifolii*– represents one of the biggest collection of cyst nematode genomes sequenced to date, utilising an Illumina NovaSeq short read sequencing platform from genomic DNA of fifty cysts per species. This is one of the first studies to employ draft *Heterodera* genomes and orthologous gene analysis to explore the genomic composition within the *Avenae* species group. The detection of *Wolbachia* in an Australian population of *H. humuli* cysts marks a significant addition to the endosymbionts associated with PPNs. The study also contributes to the growing understanding of *Wolbachia* diversity in PPNs, which has been unexplored compared to its role in filarial nematodes and arthropods.

A foundational element for gaining a comprehensive grasp of a species’ biology and evolutionary history is having a good quality reference genome. Yet, the primary obstacle persists in isolating high molecular weight DNA from organisms across the entire spectrum of life [78]. Meeting this demand for *Heterodera* spp. is particularly difficult since they are almost impossible to grow in an in vitro environment with few exceptions such as *Heterodera sacchari* that was cultured in vitro on Pluronic gel [79]. Consequently, reference genomes for cyst nematode species are typically constructed from genetic material obtained from cyst populations maintained on cultures [24–26, 40, 42–44]. This approach results in a significant level of heterozygosity in the acquired genome since the starting material contains substantial allelic variation that complicates the assembly process. The presence of diverse alleles at genomic loci hinders accurate reconstruction, leading to fragmented or misassembled sequences. When individuals are pooled together, a sequencing bias may occur, favouring certain alleles over the others [49, 80]. Addressing these challenges remains essential for obtaining accurate and representative whole genome sequences in cyst nematode species.

### Illumina short read sequencing of *Heterodera* species

The raw Illumina NovaSeq sequencing results and metrics for the six sequenced *Heterodera* species are presented in detail in Supplementary Table 4. Fastp was used for initial quality control and preprocessing of sequencing data. Five of the six *Heterodera* species were sequenced using the same parameters, with 2 × 150 bp reads, and *H. filipjevi* which was sequenced using the Illumina NovaSeq’s SP flowcell (2 × 250 bp). After filtering, the mean read lengths generally decreased across species, indicating the removal of low-quality reads or adapter sequences. Before filtering, the total number of reads varied among the species, with *H. australis* having the highest number at 196.75 million reads and *H. filipjevi* having the lowest at 19.01 million reads. A disparity in sequencing yield among the various *Heterodera* species was observed, despite comparable input DNA concentrations (Table 1). Even though the *H. mani* DNA concentration was lower than blank as detected by Quantus Fluorometer (Table 1), 50.6 million reads were generated. The quality of the remaining reads improved, as evidenced by an increase in the percentage of Q20 and Q30 bases after filtering [81]. The difference in sequencing efficiency could be due to several factors such as the potential contamination from the initial biological material, and since all cysts were field sampled, there is uncertainty associated with the age of the cysts and the inherent genomic complexities in each species. Raw reads were used for genome assembly size estimations using a kmer based approach in GenomeScope. They are reported as: *H. australis* (85.3 Mb), *H. avenae* (155.57 Mb), *H. filipjevi* (32.59 Mb), *H. humuli* (178.94 Mb), *H. mani* (123.89 Mb), and *H. trifolii* (56.01 Mb).

Quantitative metrics along with genome assembly completeness statistics of the draft assemblies are shown in Table 2. The data generated during this study was compared to an already published genome of *H. carotae* (carrot cyst nematode) NCBI GenBank BioProject accession number PRJNA774818, since the *H. carotae* cysts were similarly field sampled and the draft genome also generated from DNA extracted from a pool of fifty cysts [43]. The draft genome lengths of the six *Heterodera* genomes assembled in this study ranged from 45.4 to 116.8 Mbp with the largest genome size observed in *H. avenae*, while the smallest was found in *H. filipjevi*, despite both cyst nematode species belonging to the same *Avenae* species group [82]. This may be due to the fragmented nature of the draft genome assembly. The longest N50 value was obtained for *H. australis* (7,074 bp) followed by *H. mani* (5,925 bp), *H. avenae* (2,351 bp), *H. humuli* (1,362 bp), *H. trifolii* (1,052 bp) and *H. filipjevi* (741 bp). The disparities in sequencing performance (Supplementary Table 4), genomic coverage and the percentage of mapped genome (Table 2), possibly influenced by the starting material, highlights the need for meticulous consideration of input material in cyst nematode genomic studies. The fragmented nature of the draft genome assemblies can also be attributed to both the low starting material and the limitations of Illumina short read sequencing technology in resolving repetitive and low complexity genome regions [83]. Moreover, it is probable that natural populations of cysts collected from field soil samples exhibit elevated levels of heterozygosity, impacting both the assembly and annotation quality [80], as evidenced in the findings of this study. The use of highly accurate long-read sequencing technologies would likely generate more contiguous and haplotype-resolved assemblies in the context of complex cyst nematode genomes [47, 78, 83, 84]. This was the case for the draft genome assembly of *H. glycine* where the genome size increased from 123.84 Mb [40] to 157.9 Mb [41] following the generation of long read sequencing data.

**Table 2:** Draft genome assembly size, number of contigs, N50 value and genome completeness percentage of the six sequenced *Heterodera* species from the genomic DNA of field isolated fifty cysts compared to a published genome of *Heterodera carotae* (GenBank BioProject accession number PRJNA774818)

	Assembly statistics	H. australis	H. avenae	H. filipjevi	H. humuli	H. mani	H. trifolii	H. carotae [43]
**Minimum contig value = 500 bp**	**Common name**	Australian cereal cyst nematode	Cereal cyst nematode	Filipjev’s cereal cyst nematode	Hop cyst nematode	Ryegrass cyst nematode	Clover cyst nematode	Carrot cyst nematode
	**NCBI GenBank Genome Assembly Accession Number**	JBDLPM000000000	JBDLPL000000000	JBDLPK000000000	JBDLPJ000000000	JBDLPI000000000	JBDLPH000000000	GCA_024500135.1
	**Assembly Size (bp)**	82,886,899	116,811,496	45,419,541	116,750,469	105,775,809	100,475,064	95,118,078
	**Assembly size (Mb)**	82.8	116.8	45.4	116.7	105.7	100.4	95.1
	**Number of scaffolds**	18,642	70,665	57,668	95,680	41,671	98,793	17,839
	**Number of contigs**	18,642	70,664	57,631	95,857	41,671	98,793	17,845
	**Largest scaffold (bp)**	66,318	64,296	19,717	143,716	43,969	47,397	113,425
	**N50 value (bp)**	7,074	2,351	741	1,362	5,925	1,052	13,935
	**No. contigs > 5**,**000 bp**	5,474	3,620	160	3,201	6,115	1,140	5,030
	**No. contigs > 10**,**000 bp**	1,858	845	31	1,595	2,052	183	2,755
	**No. contigs > 25**,**000 bp**	123	53	0	294	101	2	699
	**No. contigs > 50**,**000 bp**	5	1	0	29	0	0	103
	**GC content (%)**	36.32	37.57	41.77	47.8	37.89	44.44	39.39
	**Total reads**	194,388,316	78,179,649	18,875,672	120,062,124	52,100,830	64,320,364	
	Genome Mapped %*	15.57	63.32	24.27	47.47	61.73	25.95	
	**Coverage > = 1x (%)**	99.44	98.2	92.02	93.26	99.07	89.95	
	**Average coverage depth**	31	44	9	44	37	11	
**Eukaryota_db (** ***n*** ** = 255)**	**Complete BUSCOs (%)**	165 (64.7)	108 (42.4)	14 (5.5)	173 (67.8)	164 (64.3)	69 (27.1)	149 (58.4)
	**Complete and single-copy BUSCOs (%)**	165 (64.7)	108 (42.4)	14 (5.5)	168 (65.9)	164 (64.3)	60 (23.5)	149 (0.0)
	**Complete and duplicated BUSCOs (%)**	0	0	0	5 (2.0)	2 (0.8)	9 (3.5)	0
	**Fragmented BUSCOs (%)**	29 (11.4)	72 (28.2)	99 (38.8)	29 (11.4)	39 (15.3)	115 (45.1)	52 (20.4)
	**Missing BUSCOs (%)**	61 (23.9)	75 (29.4)	142 (55.7)	53 (20.8)	52 (20.4)	71 (27.8)	54 (21.2)
**Nematoda_db (** ***n*** ** = 3131)**	**Complete BUSCOs (%)**	1468 (46.9)	973 (31.1)	128 (4.1)	1568 (50.1)	1468 (46.9)	407 (13.0)	1742 (55.1)
	**Complete and single-copy BUSCOs (%)**	1458 (46.6)	969 (30.9)	128 (4.1)	1552 (49.6)	1438 (45.9)	403 (12.9)	1702 (54.4)
	**Complete and duplicated BUSCOs (%)**	10 (0.3)	4 (0.1)	0	16 (0.5)	30 (1.0)	4 (0.1)	22 (0.7)
	**Fragmented BUSCOs (%)**	155 (5.0)	191 (6.1)	95 (3.0)	129 (4.1)	143 (4.6)	174 (5.6)	154 (4.9)
	**Missing BUSCOs (%)**	1508 (48.2)	1967 (62.8)	2908 (92.9)	1434 (45.8)	1520 (48.5)	2550 (81.4)	1253 (40.0)
	**Predicted protein coding genes using** ***H. schachtii*** **species model**	18,975	44,321	35,303	1,01,081	33,265	77,832	17,212

*% of the reads mapped back to the draft genome assembly of the respective species

n = total BUSCO groups searched

Genomic GC content ranged from 36.32% (*H. australis*) to 47.47% (*H. humuli*), which was broadly similar to *H. avenae* (37.57%), *H. mani* (37.89%), *H. filipjevi* (41.77%) and *H. trifolii* (44.44%). The average GC content for these six cyst nematodes was 40.9%, compared to the published draft genomes of *H. glycines* (36.66%), *H. schachtii* (33.23%), *H. carotae* (39.39%) and *H. filipjevi* (37%) [41–44]. Nematode genomes generally exhibit a low GC content which is attributed to evolutionary processes shaped by selective pressures and the biology of these organisms. Nematodes are often characterised by relatively small and compact genome when compared to more complex organisms [85]. The lower GC content in the six assembled genomes may contribute to structural variations, such as inversions and translocations [85]. This can impact the accuracy of bioinformatic algorithms leading to incomplete or misassembled genomic scaffold sequences within the assemblies [39]. A more accurate understanding of the GC content and its variations within and between species of nematode genomes will be realised as more whole genome data becomes available from studies that focus on enriching for nematode reads and effectively removing contamination from the assemblies.

The benchmarking universal single-copy orthologues (BUSCO) completeness scores for the final decontaminated assemblies offer a metric for assessing the quality of the draft genomes [62]. BUSCO completeness scores using the nematoda lineage for the final six decontaminated assemblies were somewhat comparable to the published genomes of *H. glycines* (59.8%) [41], *H. schachtii* (60.1%) [42], *H. carotae* (55.1%) [43] and *H. filipjevi* (55.8%) [44]. *H. humuli* (50.1%) had the highest complete BUSCO% as compared to *H. australis* (46.9%), *H. mani* (46.9%) and *H. avenae* (31.1%) using the nematode specific lineage database.

### Annotation and phylogeny

The link between genome assembly and annotation is crucial for identifying functional elements within and between the genomes [86–88]. Our study aimed to uniformly annotate the assembled draft *Heterodera* genomes to address challenges posed by highly fragmented assemblies. The bioinformatic approach presented in this study was designed to ensure that the comparative analyses and conclusions were based on the biological diversity of *Heterodera* species, rather than being influenced by the annotation methods used. Notably, the total number of predicted protein-coding genes in the six assembled draft genomes exceeded that of the comparative reference genome of *H. carotae*, which may be due to the fragmented nature of the draft assemblies (Table 2). Comparisons were made with the *H. carotae* draft genome since it was sequenced using 50 cysts which were also field sampled.

**Fig. 2:**
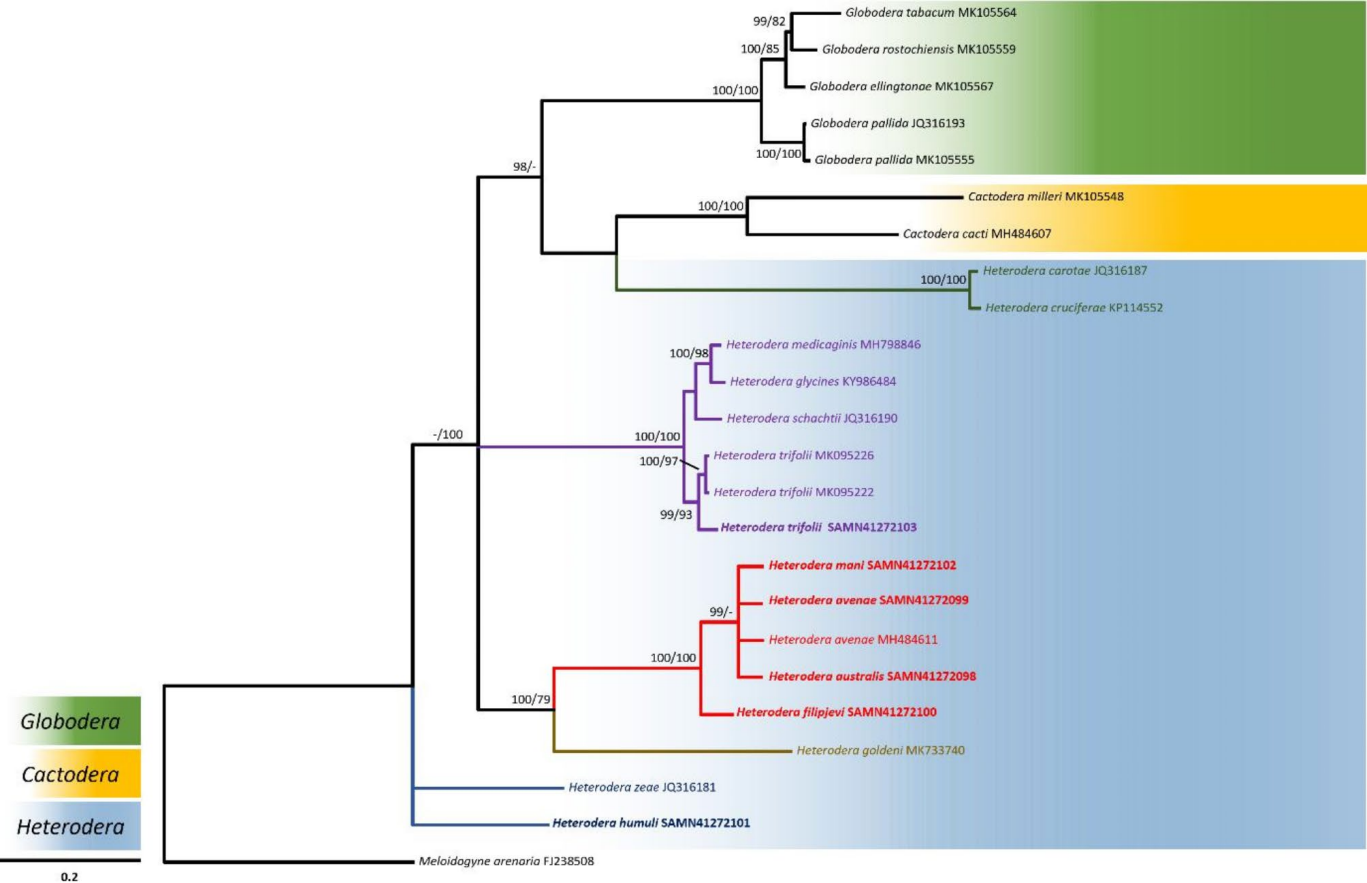
Bayesian majority-rule consensus tree of the *hsp*90 gene of the six sequenced *Heterodera* species compared to the *hsp*90 gene of the other species of the Heteroderidae family. New sequences generated during this study are highlighted in bold type. Bayesian inference (BI) posterior probabilities and maximum likelihood (ML) bootstrap support values are depicted on the nodes. Support values above 90 (pp) and 70 (bs) are shown. The scale bar indicates the number of substitutions per site. NCBI GenBank accession numbers are preceded by taxa names. Different genera are highlighted in different colours (*Globodera*: green, *Cactodera*: blue, and *Heterodera*: yellow) as shown on top of the scale bar. *Heterodera* species group branches and the associated text is shown in different colours: *Avenae* (red), *Cyperi* (Magenta), *Goettingiana* (dark green), *Humuli* (dark blue), *Sacchari* (brown), *Schachtii* (purple). *Meloidogyne arenaria* was taken as the outgroup

**Fig. 3:**
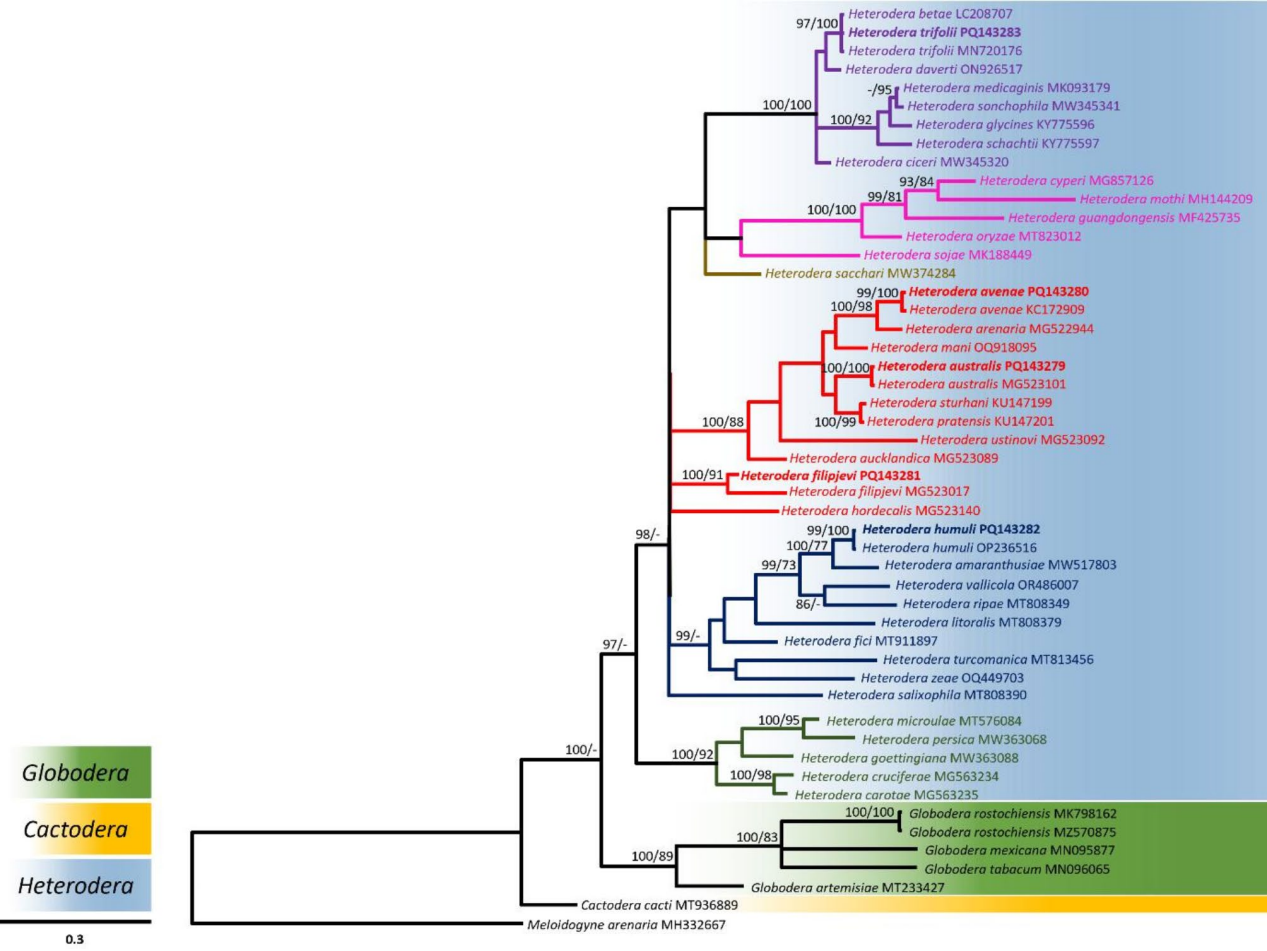
Bayesian majority-rule consensus tree of the sequenced *Heterodera* species COI gene in relation to other species of the Heteroderidae family. New sequences generated during this study are highlighted in bold type. Bayesian inference (BI) posterior probabilities and maximum likelihood (ML) bootstrap support values are depicted on the nodes. Support values above 90 (pp) and 70 (bs) are shown. The scale bar indicates the number of substitutions per site. NCBI GenBank accession numbers are preceded by taxa names. Different genera are highlighted in different colours (*Globodera*: green, *Cactodera*: blue, and *Heterodera*: yellow) as shown on top of the scale bar. *Heterodera* species group branches and the associated text is shown in different colours: *Avenae* (red), *Cyperi* (Magenta), *Goettingiana* (dark green), *Humuli* (dark blue), *Sacchari* (brown), *Schachtii* (purple). *Meloidogyne arenaria* was taken as the outgroup

**Fig. 4:**
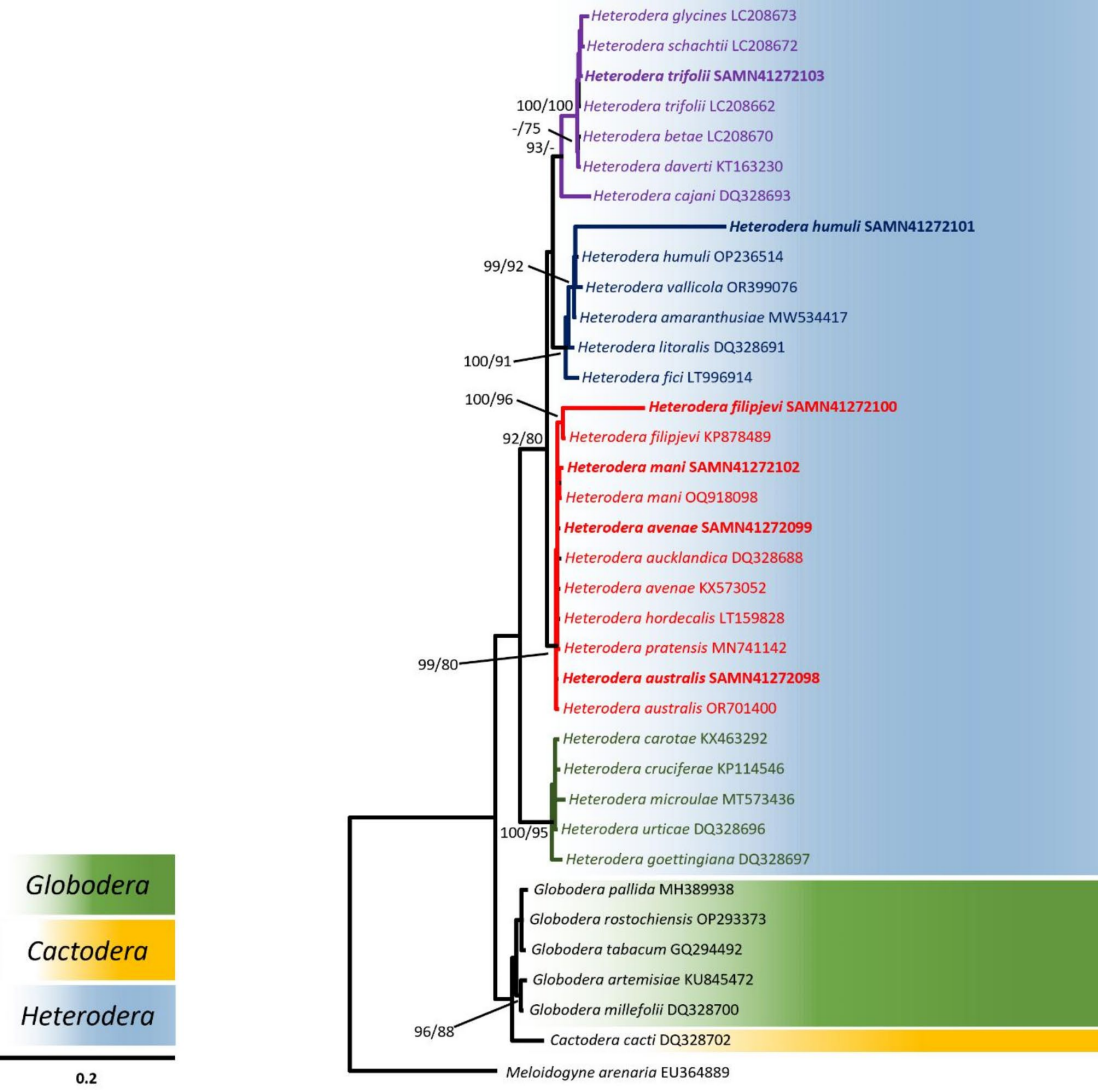
Bayesian majority-rule consensus tree of the sequenced *Heterodera* species 28 S rRNA gene sequence in relation to other species of the Heteroderidae family. New sequences generated during this study are highlighted in bold type. Bayesian inference (BI) posterior probabilities and maximum likelihood (ML) bootstrap support values are depicted on the nodes. Support values above 90 (pp) and 70 (bs) are shown. The scale bar indicates the number of substitutions per site. NCBI GenBank accession numbers are preceded by taxa names. Different genera are highlighted in different colours (*Globodera*: green, *Cactodera*: blue, and *Heterodera*: yellow) as shown on top of the scale bar. *Heterodera* species group branches and the associated text is shown in different colours: *Avenae* (red), *Cyperi* (Magenta), *Goettingiana* (dark green), *Humuli* (dark blue), *Sacchari* (brown), *Schachtii* (purple). *Meloidogyne arenaria* was taken as the outgroup

The integration of molecular approaches, including DNA barcoding and genomic sequencing, with traditional morphological taxonomy, has become pivotal in resolving complex issues related to nematode species differentiation and biosecurity concerns [89–91]. We used nucleotide sequences of the *hsp*90 (nuclear), COI (mitochondrial) and 28 S (rRNA) gene regions extracted from the assembled draft genomes for phylogenetic analysis. In this study the alignment-based method “MAAFT” [68, 69] that aligns sequence reads to a backbone alignment and places each query sequence into a backbone tree, was used. The sequence alignments were then assigned taxonomy using a phylogenetic placement [92]. The cyst-forming nematodes are classified into seven primary clades [9, 10]. Particularly close relationships were seen in this analysis between the *Avenae* and the *Sacchari* groups (Figs. 2 and 3), as well as between the *Humuli* group and the *H. salixophila* species, previously considered a member of the *Schachtii* species group (Fig. 3) [10]. The *Goettingiana* group is more closely associated to the *Globodera* genus than any other *Heterodera* species group (Figs. 2, 3 and 4). The *hsp*90 gene sequence of *H. mani* is shown as a sister taxon to *H. avenae*, implying they share a more recent common ancestor with each other than with *H. australis*. Sequences of the *hsp*90 gene from *H. carotae* and *H. cruciferae* are more closely related to the *Globodera* and *Cactodera* clades when compared to the remaining *Heterodera* species (Fig. 2). This grouping may in part be due to the absence of reference sequences available on public databases [93] for the *hsp*90 dataset necessary for a more comprehensive sequence alignment. Extensive reference specimens and sequences are required to better understand the phylogenetic relatedness of cyst nematodes, particularly for species groups that share similar morphological traits. Clear separate branching among different *Heterodera* species with strong support is shown for the phylogenetic relationship obtained using the COI dataset (Fig. 3) when compared to the phylogenetic relationships between 28 S rRNA and *hsp*90 gene sequences (Figs. 2 and 4). Lower bootstrap values in the phylogenetic trees (Figs. 2 and 4) may just be a consequence of nucleotide miscalling problems [92]. Relationships between species with low bootstrapping values involves acknowledging the uncertainty associated with those branches, considering the potential sources of errors or biases and interpreting the inferred relationships in the context of available data. Using whole genome sequencing accelerates the creation of DNA barcode databases for previously understudied organisms. Generating multi-locus datasets directly tied to physical samples substantially improves the accuracy of taxonomic classification and evolutionary analysis using DNA barcoding [94]. The genomic community can benefit from PCR-free multi-locus approaches as an initial step to fully exploit information from shotgun datasets, avoiding biases and artifacts introduced by PCR. This approach ensures the identification of genetic markers associated with virulence, host specificity and resistance for control measures [91, 92]. PCR-free methods provide cleaner, more reliable sequencing data upon the removal of redundant sequencing artefacts, facilitating accurate predictions of abundance, genomic region representation and structural variation analysis [95]. Consequently, for taxonomic phylogenetic placement in cyst nematodes, a mix of nuclear and mitochondrial gene regions should be considered [96], as utilised in this study.

The *Avenae* species group is one of the largest within the *Heterodera* genus exclusively parasitising monocotyledonous plants [82]. Currently, 12 species belong to the *Avenae* group: *H. latipons*, *H. hordecalis*, with the remaining ten forming the *H. avenae* complex (*H. arenaria*,* H. avenae*,* H. aucklandica*,* H. australis*,* H. filipjevi*,* H. mani*,* H. pratensis*,* H. riparia*,* H. sturhani* and *H. ustinovi*). Five species within the *Avenae* group are recognised as significant agricultural pests, collectively termed as cereal cyst nematodes (CCN) [97]. Four of these CCNs– *H. avenae*,* H. filipjevi*,* H. sturhani* and *H. australis*– cause substantial economic damage to cereal crops in various grain cropping regions [98, 99]. In contrast, the other six species in the *H. avenae* complex primarily parasitise less economically important grasses [82]. However, biosecurity measures should be implemented for all members of the *H. avenae* group that have not been recorded in Australia as the possibility of these parasites evolving to infect new hosts cannot be excluded [21]. This study sequenced and assembled draft genomes of four *Heterodera* species belonging to the *Avenae* species group, three of which– *H. australis*,* H. avenae* and *H. filipjevi* are of particular importance to the Australian plant biosecurity system [21]. Cereal cyst nematode (CCN) has been present in Australia since the 1930s, causing significant losses in Australia’s cereal growing regions [100]. Historically, Australian CCN was considered to be *Heterodera avenae*. However, in 2002, *Heterodera australis* was proposed as a distinct species based on biochemical and molecular differences [101]. There has been ongoing debate about the validity of this new species and whether it is native to Australia [100, 101] until recently, Huston, Khudhir et al. [21] concluded that *H. avenae* is absent from Australia and the study supported the validity of *Heterodera australis* as the name for the Australian CCN. Moreover, their study also suggested that *H. australis* is not native to Australia and was likely introduced from Asia in the 1850s, rather than from Europe. This hypothesis was speculated using archival and newly collected cysts infested soil samples from locations across Australia. This also suggested that the resistance breeding that was introduced the early 90s was potentially for varieties that were susceptible to *H. australis* instead of *H. avenae.*

**Fig. 5:**
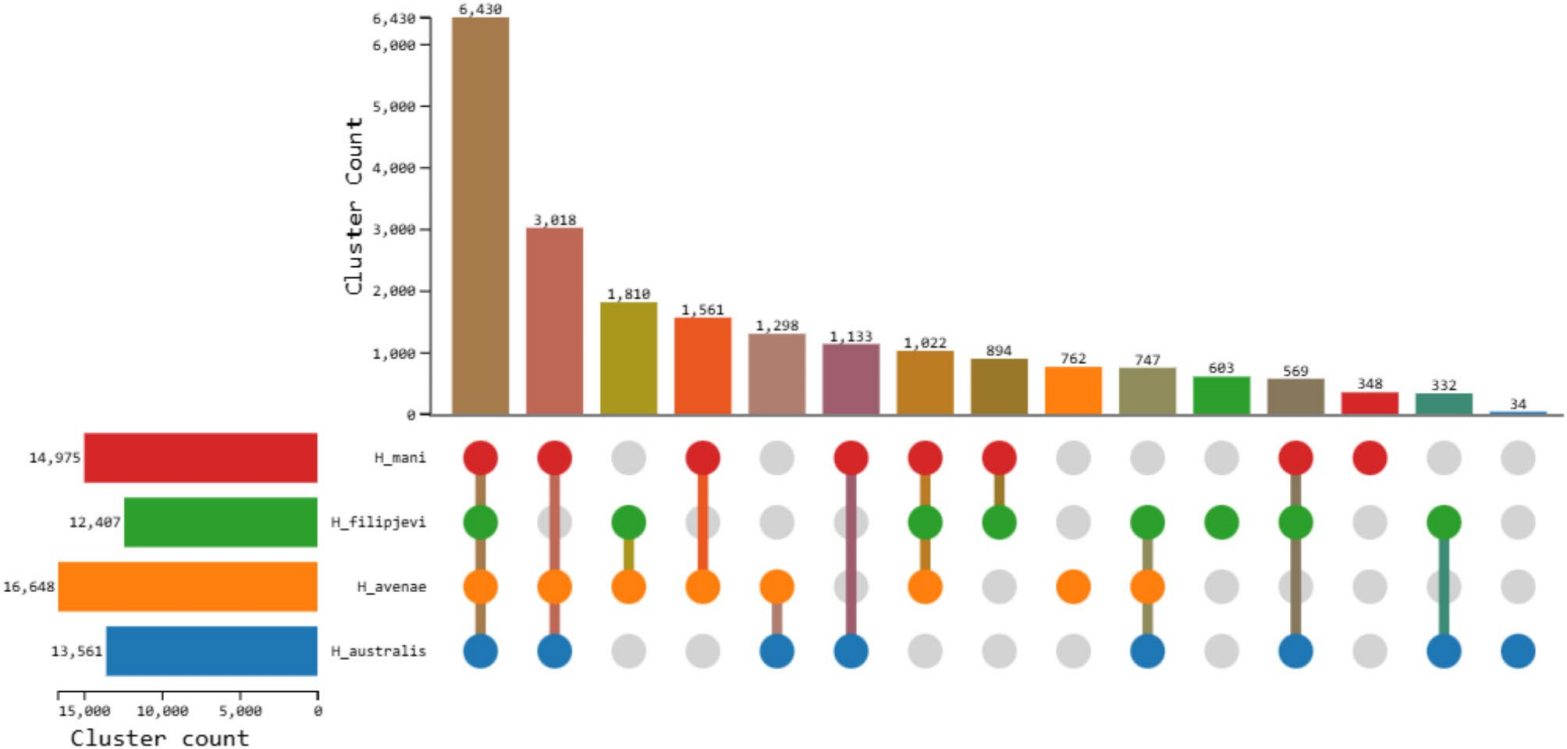
UpSet chart presenting the number of orthologous gene clusters in each species of the *Avenae* group as well as the number of unique and shared homologous clusters among *H. australis* (blue), *H. avenae* (orange), *H. filipjevi* (green) and *H. mani* (red). The bar chart on the bottom left indicates the cluster count representing the number of clusters present in each of the *Heterodera* species

**Fig. 6:**
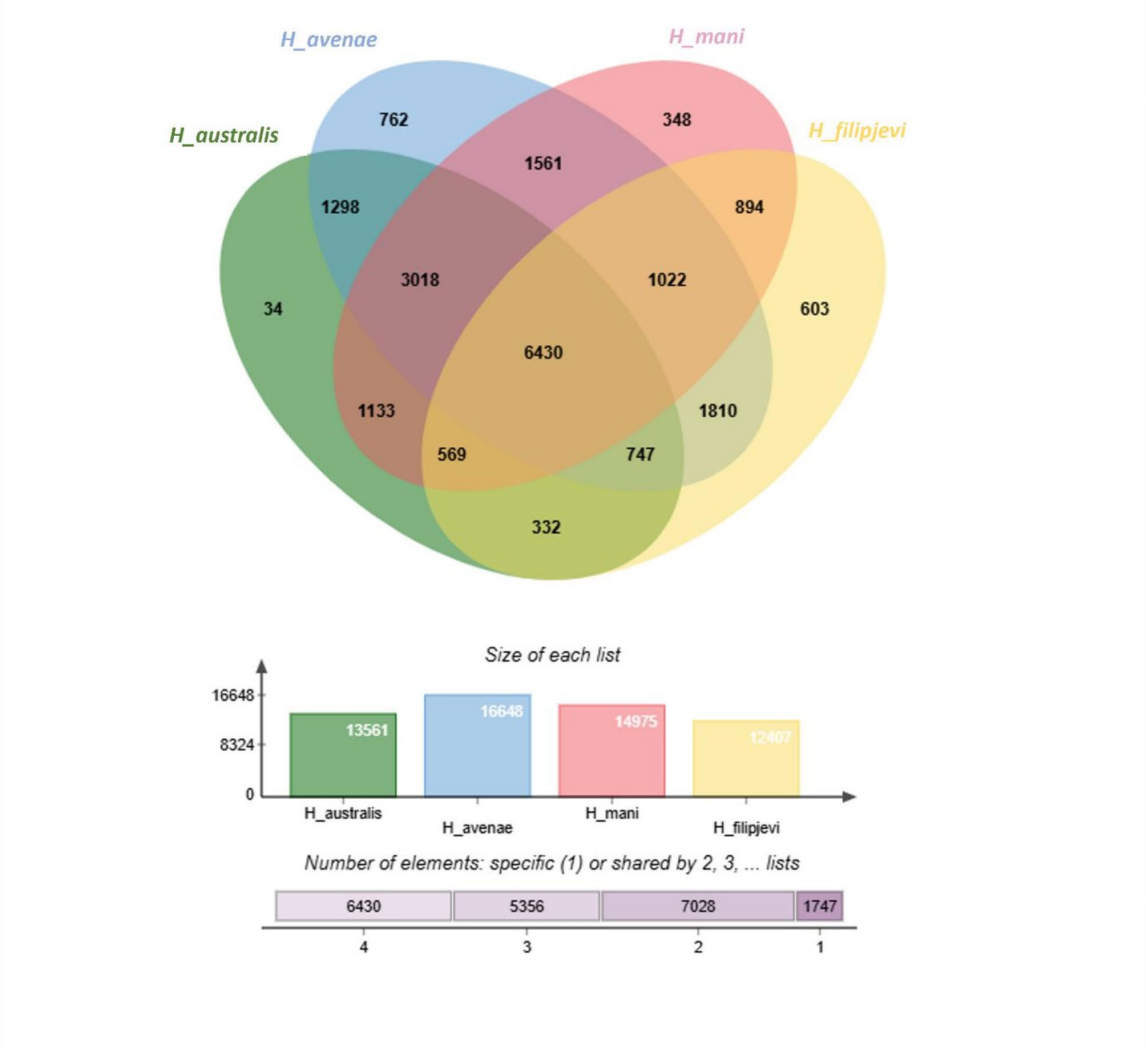
Venn diagram of orthologous gene clusters present in *Heterodera* species– *H. australis*,* H. avenae*,* H. filipjevi* and *H. mani* belonging to the *Avenae* species group of *Heterodera* genus. The bar chart on the lower end of the image shows the size of the protein clusters in each species and the cumulative numbers of the shared elements based on the Venn diagram

To explore the genomic landscape within the *Avenae* species group, this study looked at the composition of orthologous gene clusters using OrthoVenn3. Using the generated draft genome data, a total of 14,975 orthologous gene clusters were found in *H. mani*, 12,407 in *H. filipjevi*, 13,561 in *H.* australis, and 16,648 in *H. avenae* (Fig. 5). In total, 6,430 orthologous gene clusters were common across the four *Avenae* complex species (Fig. 6). A further 1,133 orthologous gene clusters were shared between *H. australis* and *H. mani*. *H. mani* and *H. avenae* shared 1,561 orthologous gene clusters while 1,298 orthologous gene clusters were shared between *H. australis* and *H. avenae* (Fig. 6). The three species together share 3,018 orthologous gene clusters. Both *H. avenae* and *H. australis* share more orthologous gene clusters with *H. mani* than with each other. The findings suggested that *H. mani* is either more closely related to *H. australis* than to *H. avenae*, or it is equally related to both, a finding that aligns directly with the phylogenetic analyses of Huston, Khudhir et al. [21]. *H. avenae* (762) followed by *H. filipjevi* (603) had the highest number of orthologous gene clusters that are not shared with the other two *Heterodera* species. This discourse is also evident in the phylogenetic analysis of the *hsp*90 and COI nucleotide gene sequences (Figs. 2 and 3) where *H. filipjevi* falls in a separate branch with a high bootstrap supporting value for both maximum likelihood and Bayesian inference. Future studies could delve into the phylogenetic relationships between different *Heterodera* species and its species groups to better understand the dynamics and functional relationships.

### Metagenome analysis of the sequenced *Heterodera* fifty cysts

Cysts sampled from field soil samples in their native habitats or host plants carry a species-rich bacterial community, primarily characterised by the prevalence of Proteobacteria, as well as Actinobacteria, Bacteroidetes, Ascomycota, Firmicutes, Chordata, and Planctomycetes (Supplementary Table 5). Sequencing the metagenome of *Heterodera* species cysts was an inevitable consequence of the input sample that aimed to sequence the host cyst genome. To analyse the cyst microbial metagenome the initial no-hit scaffolds from the BlobTool analysis (Supplementary Fig. 1) were retained and further analysed via the Kraken 2 database to filter any potential fungal and bacterial contaminant sequences missed by the BlobTools analysis (Supplementary Table 1). The removal of scaffolds and read pairs associated with metagenomic contaminants ensured the reliability of *Heterodera* genomic data, reinforcing the accuracy of subsequent comparative analyses, and concentrated the microbial sequence reads for further analyses.

**Fig. 7:**
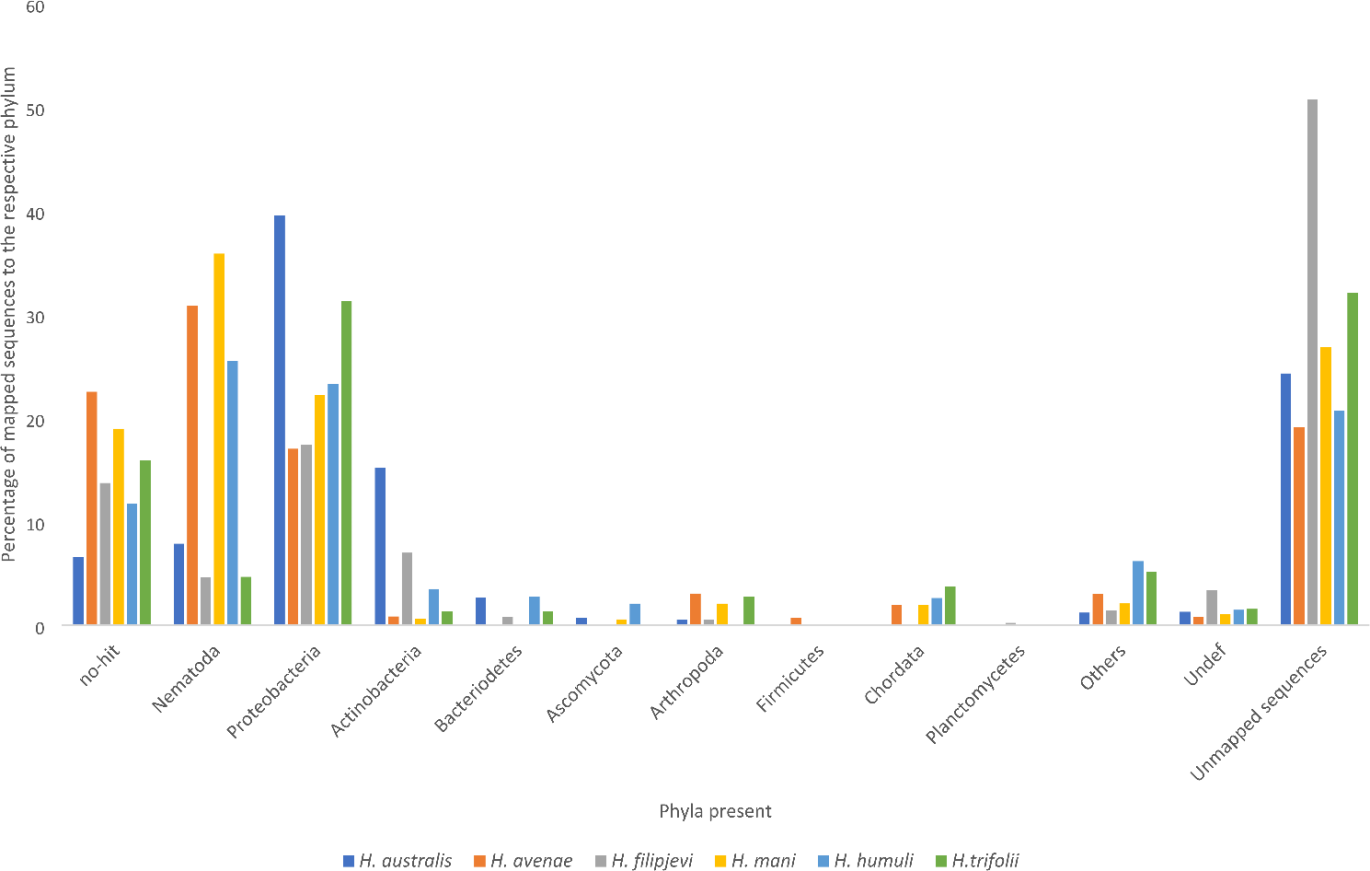
The distribution of different phyla identified in the metagenomic samples of the assembled *Heterodera* species– *H. australis*,* H. avenae*,* H. filipjevi*,* H. humuli*,* H. mani* and *H. trifolii*. Each bar represents the proportion of sequences mapped and assigned to the respective phylum in the metagenome of the fifty cysts. The bars grouped furthest on the right represent the percentage of unmapped sequences as identified by BlobTool analysis. The number of taxonomic groups plotted in the BlobPlot is ‘7’ and remaining groups are binned into the category ‘Others’. Absence of a node at the taxonomic rank, bins the sequences into the ‘Undef’ category

Microorganisms are increasingly used for potential biocontrol strategies in field, and a diverse array of bacteria and fungi have demonstrated the potential to mitigate nematode infection in plants, either through direct or indirect mechanisms [102–106]. The integration of high-throughput sequencing, taxonomic annotation, and metagenomic analysis provided a robust framework for unravelling the intricate genomic landscapes of nematodes and their microbial associates. Entomopathogenic nematodes form mutualistic relationships with *Photorhabdus* and *Xenorhabdus* bacteria, which aid in insect infection. While not obligate, these bacteria reside in juvenile nematodes and are released into insect hemolymph, causing septicemia. Their genomes encode insecticidal toxins, molecules supporting nematode development, and proteins suppressing immune defences and microbial competitors, showcasing dual functionality [107]. Endosymbiont relationship with other parasitic nematodes have been largely studied [108–111], however, sparse resources are available on the microbial communities associated with cyst nematodes [112–115]. We identified a varied bacterial community from the sequenced cysts we collected from native environments or host plants with Proteobacteria, the dominant phylum identified in *H. australis* (39.56%), *H. filipjevi* (17.43%) and *H. trifolii* (31.3%) (Fig. 7).

The abundance of Actinobacteria contigs in our sequenced dataset varied significantly, with *H. australis* having the highest prevalence at 15.19% and *H. avenae* the lowest at 0.86%. Bacteroidetes and Ascomycota had lower percentages across all species, while Arthropoda and Firmicutes were more variable, with notable peaks in *H. avenae* and *H. humuli*. Cyst nematodes are natural reservoirs of microorganisms and this can be attributed to their prolonged existence in soil and the distinct environmental conditions within and around the enclosed space of cysts, where a variety of bacteria remain concealed, and the presence of bacterial and fungal contigs in the assembly represent the microbiome that is associated with the sequencing of cyst nematodes [112]. This association of bacteria and fungi maybe subjective depending on the quality of cysts. The data hence generated in future studies can investigate microbiomes associated with *Heterodera* species and their potential significance.

The sequencing data from all sequenced microbial species revealed that all the longest scaffolds were composed of bacterial sequences (data not shown). The presence of *Wolbachia* sequences were identified at the initial screening of the bacterial sequences from the metagenomic sequences that were binned to enable the assembly of the draft genome of *H. humuli*. The *Wolbachia* isolate obtained from *Helicotylenchus* species (designated as *w*Tex, NCBI GenBank accession number GCA_022836975.1) [75] served as the reference genome for extracting *Wolbachia* reads from the *H. humuli* dataset. While *Cardinium*, another endosymbiont has been reported in cyst nematodes [116], the presence of *Wolbachia* in Australian *H. humuli* genome data represents a novel finding. The *Wolbachia* was subsequently designated as *Wolbachia* sp.– endosymbiont of *Heterodera humuli* (isolate: *w*Hhum). The assembled genome size of the isolate *w*Hhum was 807.7 Kbp consisting of 449 contigs having a GC content of 34.98% (Table 3) and the genome sequence deposited as GenBank accession number JBGGJS000000000.

**Table 3:** Comparative genome features of the newly generated draft genome assembly of *Wolbachia* from Australian *Heterodera humuli* cyst population (isolate: *w*Hhum) during this study and its closest relatives

Host (common name)	Wolbachia Isolate name	Genome assembly length (bp)	%GC	Contigs	NCBI accession
*Heterodera humuli* (hop cyst nematode), Australia	*w*Hhum	807,726*	34.98	449	GCA_042096835.1 (This study)
*Heterodera humuli* (hop cyst nematode), USA	*w*Hhum	1,051,007	32.6	1	SAMN40188821
*Helicotylenchus* sp.	*w*Tex	1,013,022	33.49	192	GCA_022836975.1
*Pratylenchus penetrans* (root lesion nematode)	*w*Ppe	975,127	32.16	36	GCA_001752665.1
*Pentalonia nigronervosa* (banana aphid)	*w*Pni	1,457,187	34	187	GCA_014534705.1
*Folsomia candida* (springtail)	*w*Fol	1,801,626	34	1	GCA_001931755.2
*Ctenocephalides felis* (cat flea)	*w*CfeT	1,495,538	35	1	GCA_012277295.1
*Cimex hemipterus* (bedbug)	*w*Chem	28,394	33.5	56	GCA_003704325.1

*Minimum contig value = 200 bp

Mutualistic relationships between bacteria and nematodes have been observed in the interaction between the endosymbiont *Wolbachia* and certain other filarial nematodes from the Onchocercidae family, which includes medically significant parasites affecting humans and animals [117]. *Wolbachia*, a type of alpha-proteobacterium closely related to *Ehrlichia*, *Anaplasma* and *Rickettsia*, is commonly found as a parasitic associate in insects and other arthropods [118]. However, in nematodes it has evolved into a mutualistic role and was thought to be limited to a specific subgroup within the filarial nematode family Onchocercidae, with *Wolbachia* having the potential to introduce antibiotics as treatment for filarial diseases as one of the most significant implications of discovering this mutualistic behaviour [119]. Efforts to detect *Wolbachia* in non-filarial nematodes have been unsuccessful [120], except for some instances where *Wolbachia* sequences were detected in the plant parasitic burrowing nematode, *Radopholus similis* [121, 122]. *Wolbachia* has also been identified in *Pratylenchus penetrans* (isolate: *w*Ppe) [122, 123] and *Helicotylenchus* species (isolate: *w*Tex) [75]. Recently, the presence of *Wolbachia* in *Heterodera humuli* (isolate: *w*Hum) from a cyst population from Oregon, USA was identified which was sequenced using PacBio long read sequencing technology [124].

**Fig. 8:**
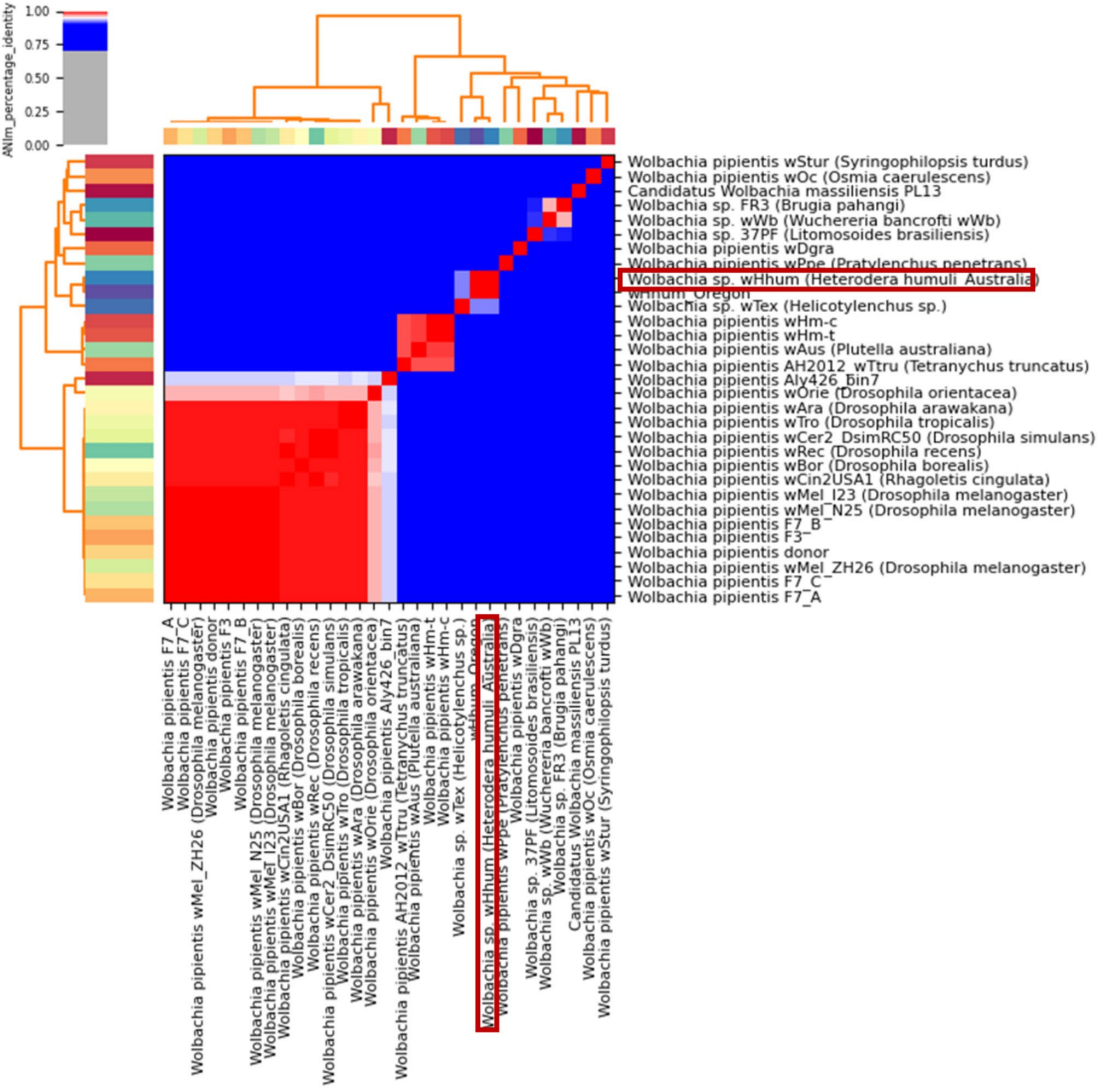
Comparison of the average nucleotide identity (ANI) values between whole genome sequences of 32 *Wolbachia* isolates from NCBI GenBank and the newly generated *Wolbachia* isolate *w*Hhum: Endosymbiont of *Heterodera humuli* (highlighted in the red box). ANI analysis based on MUMmer alignment of the genome sequences was performed using pyANI. Red squares represent ANI values of 96% or higher

To further assess the phylogenetic relationship between the Australian *w*Hhum isolate and other closely related *Wolbachia* species, we computed the average nucleotide identity (ANI) [76] between coding regions of *Wolbachia* genomes associated with various nematode and insect species available on NCBI GenBank (Supplementary Table 3). Typically, ANI values above 96% indicate the same species [76]. Our analysis showed that the ANI values shared between different *Wolbachia* isolates and the generated *Wolbachia* genome from *H. humuli* ranged between 80 and 92% (Supplementary Table 6) except for 99.6% identity with the *w*Hhum isolate from USA. A cluster dendrogram (Fig. 8) from the ANI analysis showed that the Australian *w*Hhum isolate grouped together with the *Wolbachia* endosymbiont of *Heterodera humuli* (from USA) [124], *Pratylenchus penetrans* (*w*Ppe) [123] and *w*Tex [75] isolates. Notably, they formed a distinct branch, indicating a close relationship while also maintaining a separate species identity. The *w*Hhum (USA), *w*Tex and *w*Ppe isolates shared 99.6%, 92.4% and 83.6% average nucleotide identity with the newly extracted Australian *w*Hhum isolate respectively. These findings show that Australian *w*Hhum might be a different strain of *Wolbachia* endosymbiont from the data obtained after sequencing *H. humuli* cysts collected from Oregon, USA.

The reported genome size of *w*Hhum (807.7 Kb) aligned with a common characteristic of relatively small genomes observed in *Wolbachia* species, pointing towards a pattern of genome reduction that is a characteristic of endosymbiotic bacteria [125, 126]. This reduction in genome size is often attributed to the evolutionary adaptation of *Wolbachia* to its symbiotic lifestyle within the nematode hosts. In such relationships, symbionts tend to lose non-essential genes since the host provides a protected environment and highlights the specialized and co-evolutionary nature of the relationship between *Wolbachia* and its nematode hosts [127].

This is the first recorded evidence of *Wolbachia* from a cyst nematode species using short read sequencing data. *Wolbachia* sequences were only seen in the genome data of *H. humuli* and not the other five sequenced cyst nematode species during this study and additional research efforts are needed to provide a better understanding of this endosymbiotic association. The significance of this discovery extends beyond the taxonomic classification of the identified *Wolbachia* isolate. It opens avenues for exploring the functional roles of *Wolbachia* in the context of nematode biology. These intracellular microbes are widespread in nature and are particularly intriguing for their ability to manipulate the reproduction and physiology of their hosts. Cyst nematodes exhibit environmental sex determination, where external factors influence whether an individual develops as male or female. For example, environmental cues such as nutrient availability, host plant condition, and population density can impact the proportion of males and females within a population. This adaptive strategy allows cyst nematodes to optimize reproductive success under varying environmental conditions [128]. This strategy may well be regulated due the presence of endosymbionts such as *Wolbachia*, future studies can investigate deeper into this mechanism. The understanding of nematode and their endosymbionts may also be applied to nematode management strategies through adopting them as biological controls. The genomic characterisation, taxonomic classification, and comparative analyses provide a foundation for future research exploring the functional implications of *Wolbachia* in cyst nematodes.

## Conclusion

Genomic research on cyst nematodes represents a cutting-edge field with far reaching implications for agriculture, biology, and the development of control strategies and management. We conclude that fifty cysts are a relevant sample size for sequencing draft *Heterodera* genomes that contain valuable information to serve diagnostic and Australian biosecurity purposes, while also providing information on their associated microbiomes. The draft genomes generated in this study provide a baseline for further investigation into the basic biology of *Heterodera* species and a resource for the greater nematology community. Future sequencing using long read technology will improve the draft genome quality and enhance their usefulness, however, in its current state these genomes offer researchers a resource for development of diagnostic markers for rapid species identification and to examine genomic similarities across different species groups of the genus. Moreover, a comparative analysis of the genomes and the associated effectors of *Heterodera* species with those of other PPNs has the potential to enhance research on evolutionary and lifestyle mechanisms. Despite the inherent challenges associated with sequencing *Heterodera* species, the imperative to sequence these species lies in the potential for transformative discoveries that can address critical global challenges related to food security, sustainable agriculture and biodiversity conservation in the face of threats posed by potential cyst species targeting Australian native flora.

## Electronic supplementary material

Below is the link to the electronic supplementary material.

Supplementary Material 1

## Data Availability

This Whole Genome Shotgun Sequencing project has been deposited at DDBJ/ENA/GenBank under BioProject PRJNA1109461.

## Abbreviations

PPN: Plant parasitic nematode
WGS: Whole genome sequencing
NCBI: National Center for Biotechnology Information
BLAST: Basic Local Alignment Search Tool
NPPP: National Priority Plant Pests
DAFF: Department of Agriculture, Fisheries and Forestry
BUSCO: Benchmarking Universal Single-Copy Orthologues
QUAST: Quality Assessment tool for Genome Assemblies
ANI: Average Nucleotide Identity
CCN: Cereal cyst nematode
Bp: Base pairs
G: Guanine
C: Cytosine
Mbp: Mega base pairs
BI: Bayesian Inference
ML: Maximum Likelihood
hsp90: Heat shock protein 90
mtCOI: Mitochondrial cytochrome c oxidase I
28S rRNA: Large subunit ribosomal RNA
