# Supplementary material for: Insights from draft genomes of Heterodera species isolated from field soil samples
Source: BMC Genomics. 2025 Feb 18;26:158. doi: 10.1186/s12864-025-11351-0 (PMC11834393; doi:10.1186/s12864-025-11351-0)
Supplement: Supplementary file 1 — Supplementary Material 1 [file 12864_2025_11351_MOESM1_ESM.docx]

**Supplementary Figures**

**
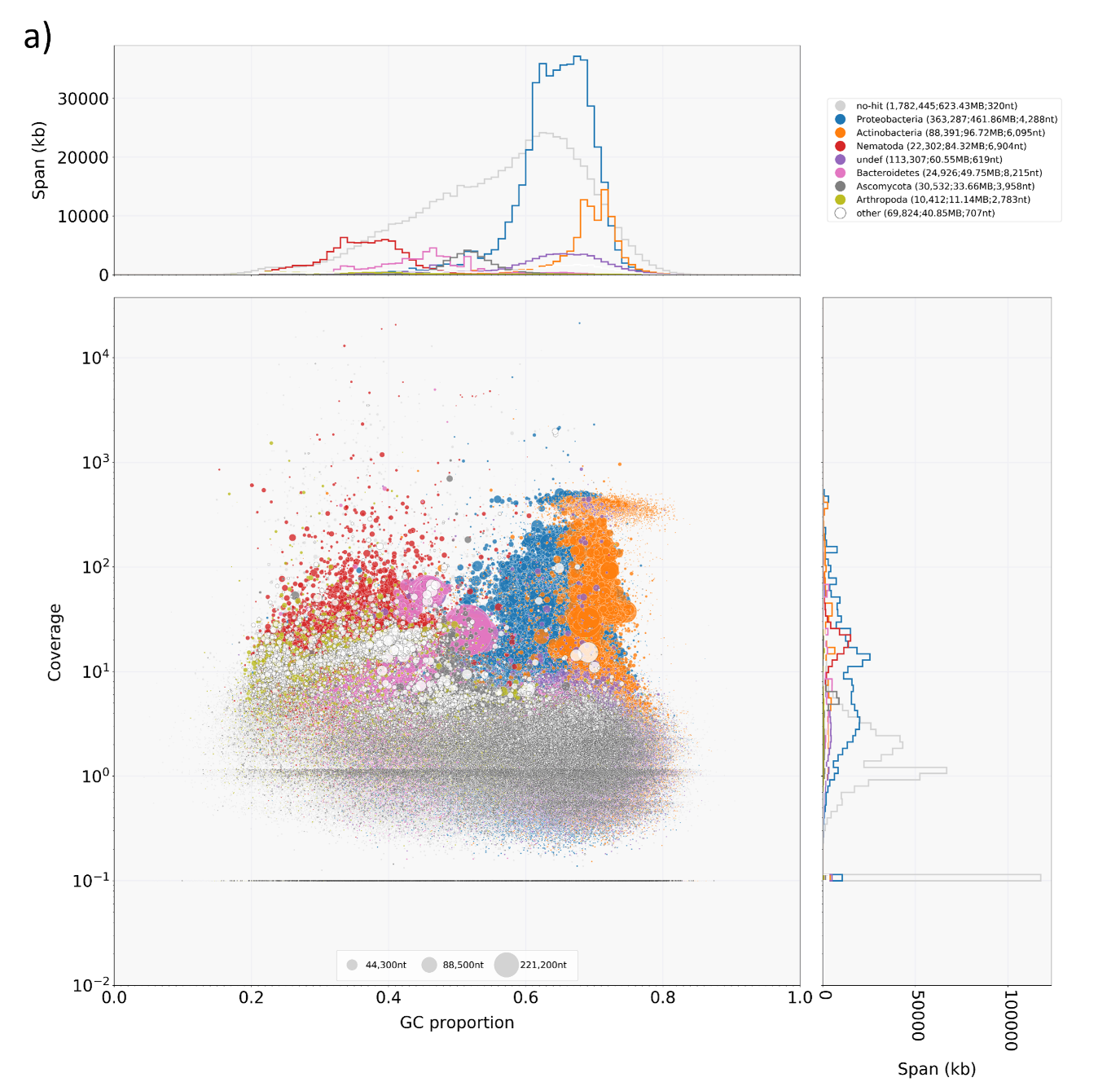
**

**
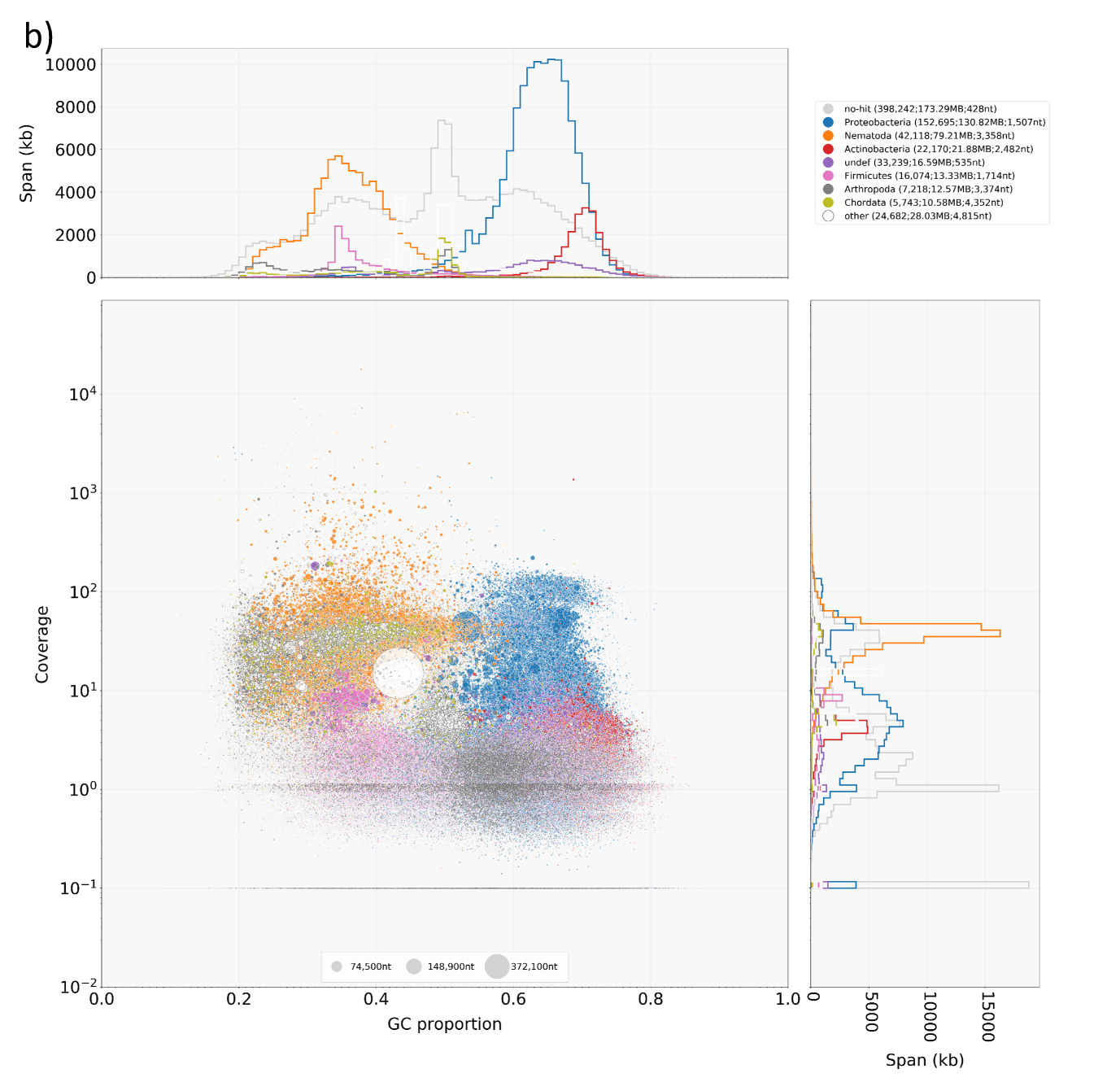
**

**
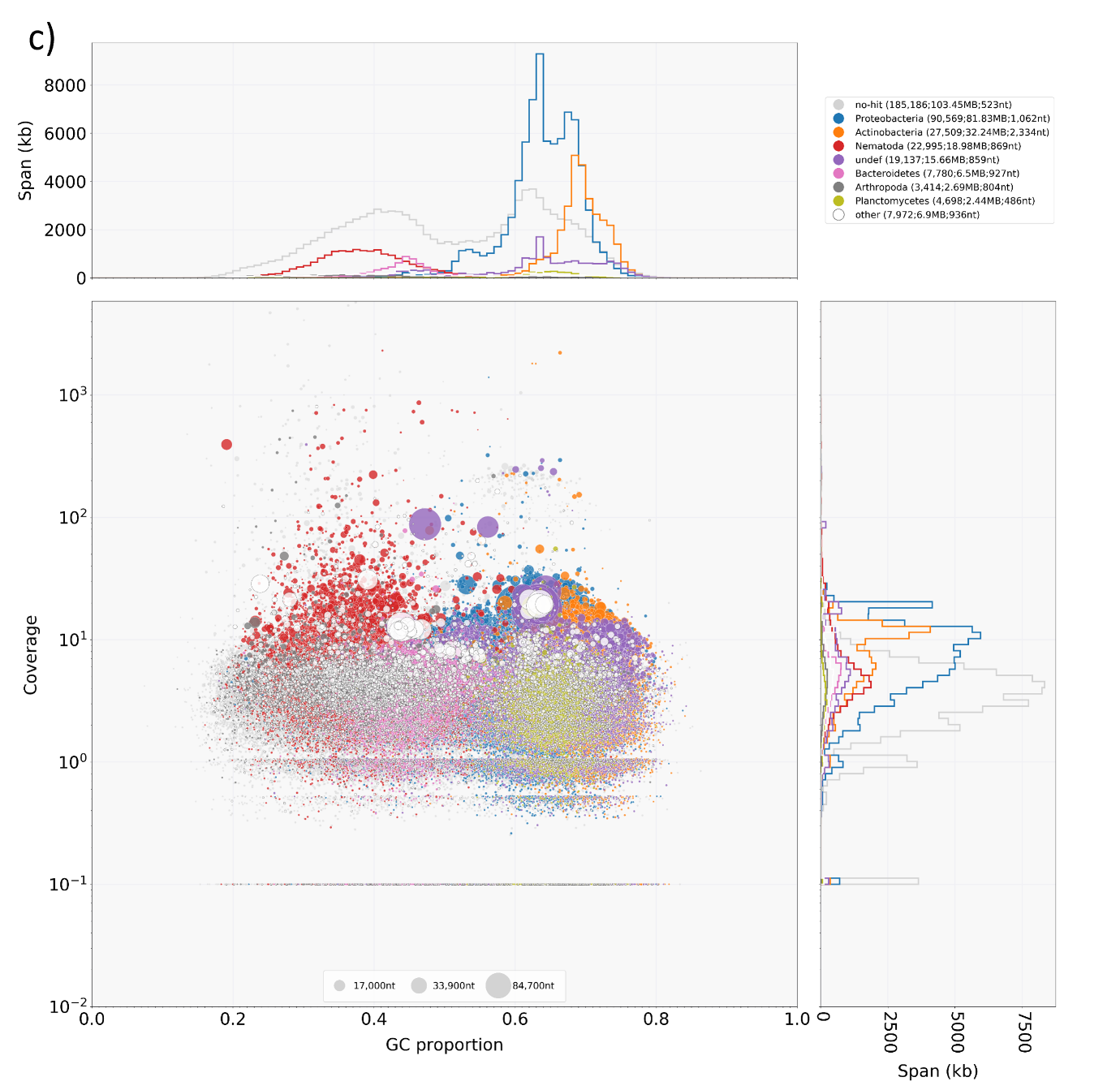
**

**
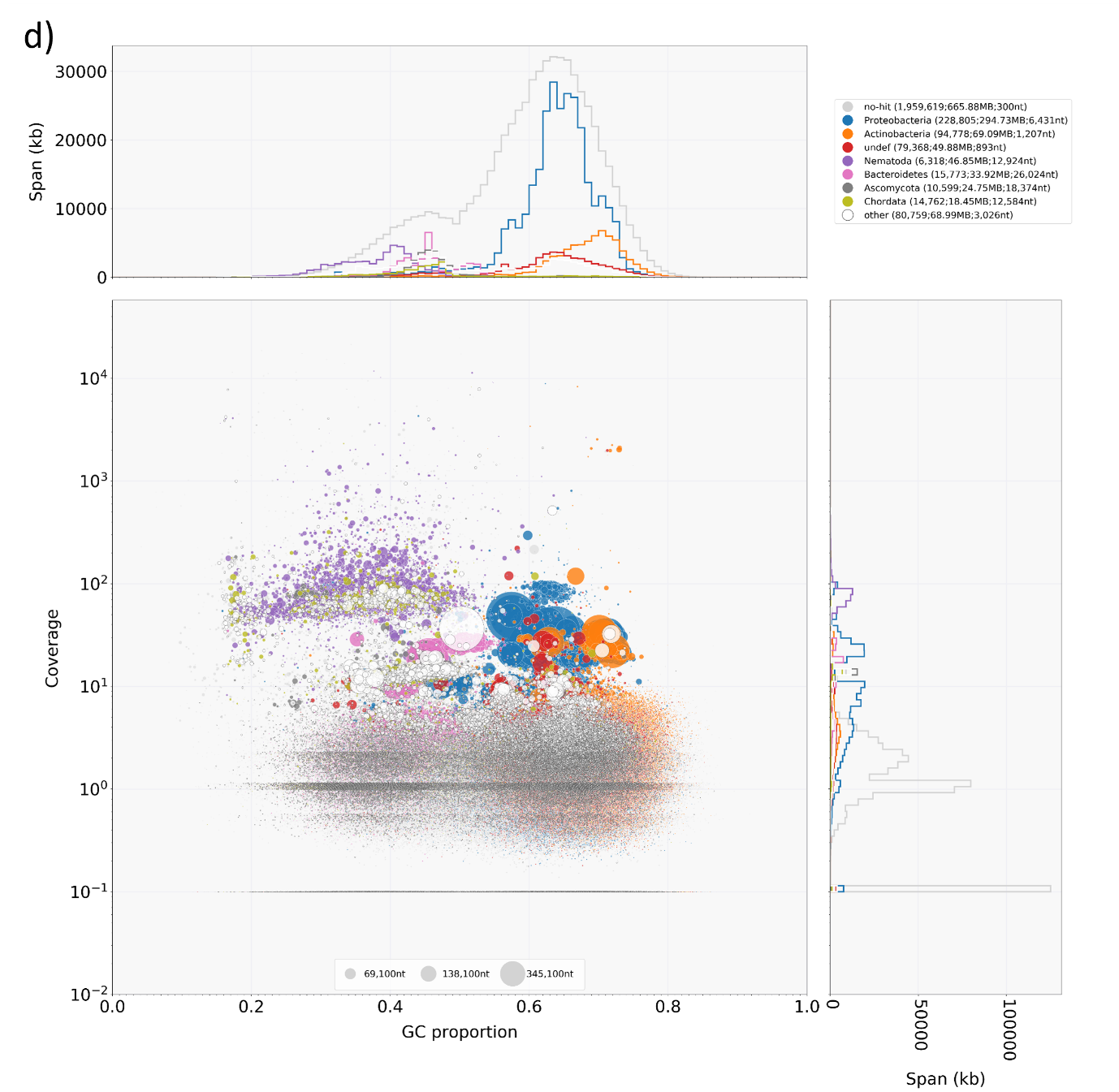
**

**
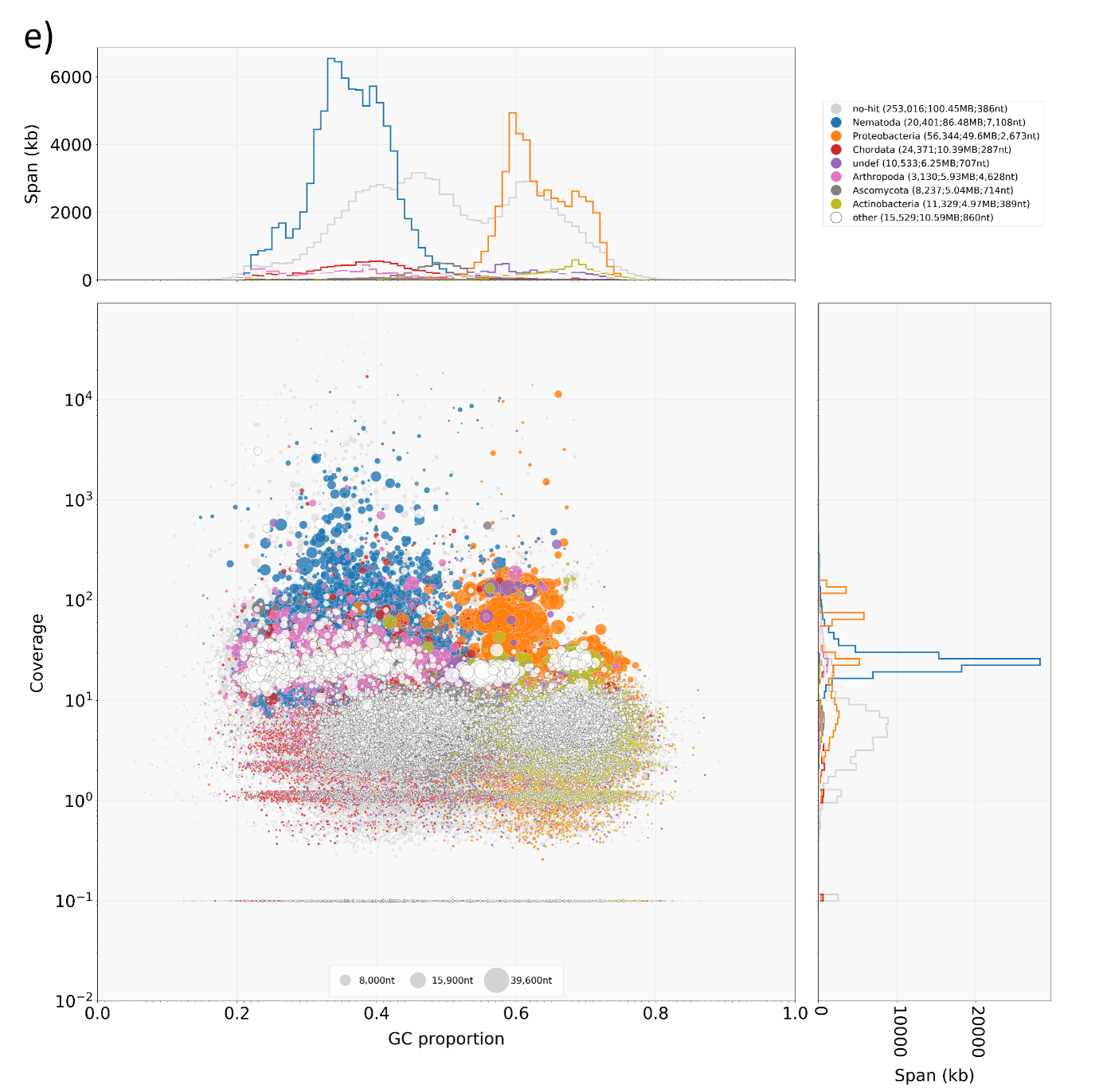

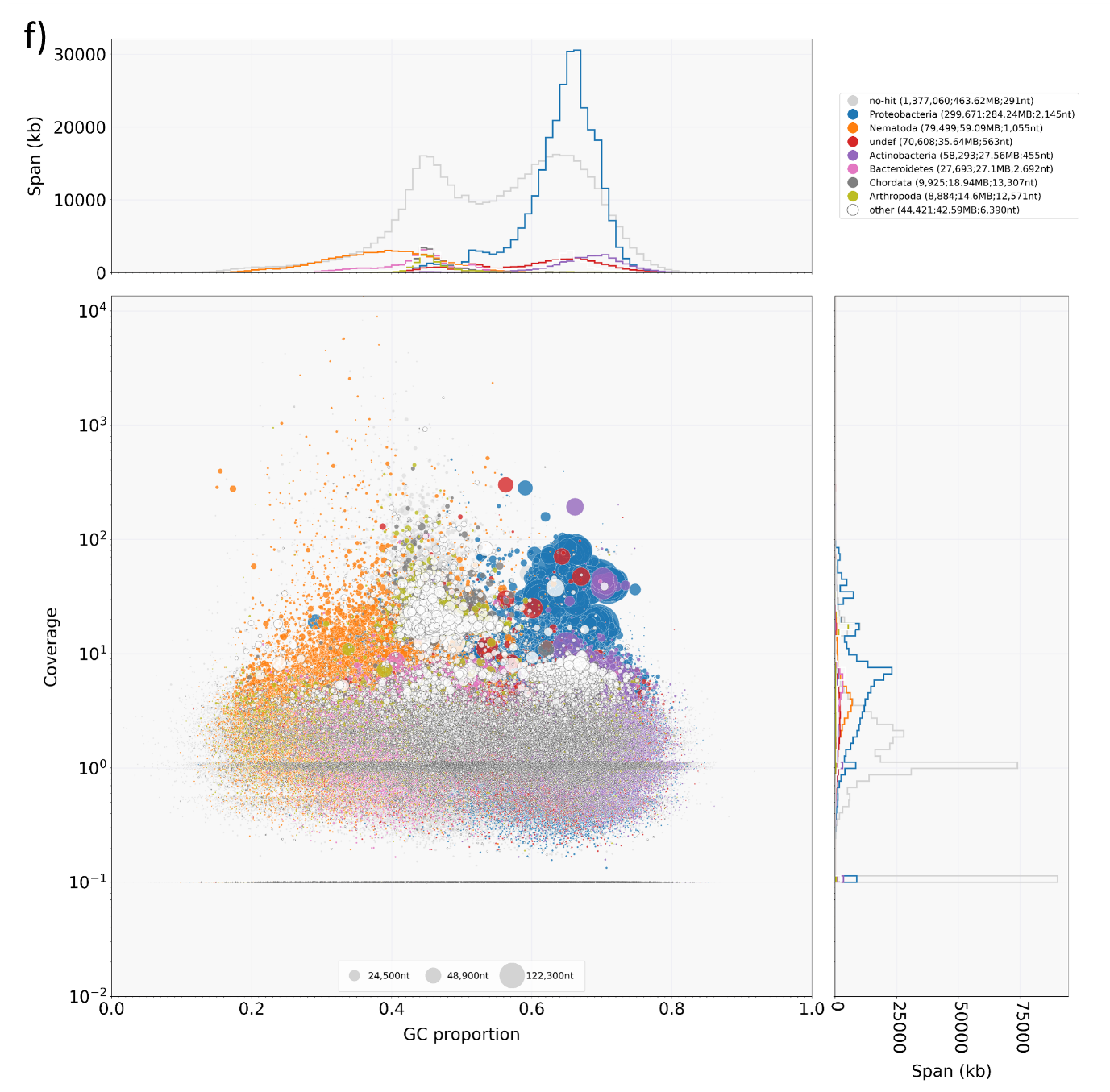
**

**Supplementary Figure 1** BlobPlots of the draft *Heterodera* genome assemblies; a) *H. australis*, b) *H. avenae*, c) *H. filipjevi*, d) *H. humuli*, e) *H. mani* and f) *H. trifolii* before removing contamination. Each circle is a contig proportionally scaled by contig length and coloured by taxonomic annotation based on BLAST similarity search results. Contigs are positioned based on the GC content (X- axis) and the coverage of Illumina NovaSeq reads (Y-axis). There are some contigs of Proteobacteria and Actinobacteria origin at high GC and variable coverage indicating potential contamination. These contigs along with other non-nematode scaffolds were removed from the assembly. The legend on the top right corner of each plot provides a coloured representation of the identified taxonomic groups.

**Supplementary Tables**

**Supplementary Table 1** List of unclassified sequences obtained each from sequenced *Heterodera* species each after decontamination steps using the Kraken 2 bacterial and fungal database. The initial no-hit sequences were identified using BlobPlot taxonomic assignment

| **Species** | **Total no-hit sequences** | **Bacterial db** | | **Total unclassified bacterial sequences** | **Fungal db** | |
| --- | --- | --- | --- | --- | --- | --- |
|  |  | **Classified** | **Unclassified** |  | **Classified** | **Unclassified** |
| *H. filipjevi* | 208,181 | 56,438 | 151,743 | 151,743 | 461 | 151,282 |
| *H. trifolii* | 1,377,060 | 264,849 | 1,112,211 | 1,112,211 | 3,748 | 1,108,463 |
| *H. avenae* | 398,242 | 100,255 | 297,987 | 297,987 | 5,649 | 292,338 |
| *H. humuli* | 1,959,619 | 365,042 | 1,594,577 | 1,594,577 | 3,377 | 1,591,200 |
| *H. mani* | 253,016 | 43,915 | 209,101 | 209,101 | 1,781 | 207,320 |
| *H. australis* | 151,716 | 57,210 | 94,506 | 94,506 | 989 | 93,517 |

**Supplementary Table 2** Details of the extracted gene sequences and their associated NCBI GenBank Accession numbers including the BioProject number, BioSample details and NCBI submission ID

| **NCBI Submission ID** | **Organism** | **Isolate** | **BioProject** | **BioSample** | **GenBank accession** | **COI accessions** |
| --- | --- | --- | --- | --- | --- | --- |
| SUB14431862 | *Heterodera trifolii* | ACT_H_tri | PRJNA1109461 | SAMN41272103 | JBDLPH000000000 | PQ143283 |
| SUB14431862 | *Heterodera mani* | Tas_H_mani | PRJNA1109461 | SAMN41272102 | JBDLPI000000000 |  |
| SUB14431862 | *Heterodera humuli* | Tas_H_hum | PRJNA1109461 | SAMN41272101 | JBDLPJ000000000 | PQ143282 |
| SUB14431862 | *Heterodera filipjevi* | NP_H_fil | PRJNA1109461 | SAMN41272100 | JBDLPK000000000 | PQ143281 |
| SUB14431862 | *Heterodera avenae* | HP_H_av | PRJNA1109461 | SAMN41272099 | JBDLPL000000000 | PQ143280 |
| SUB14431862 | *Heterodera australis* | SA_H_aus | PRJNA1109461 | SAMN41272098 | JBDLPM000000000 | PQ143279 |

**Supplementary Table 3** NCBI GenBank Assembly accessions of *Wolbachia* genome isolates used for the generation of the average nucleotide percentage similarity plot. These sequences were obtained from NCBI GenBank. The yellow highlighted box represents the isolate assembled during the present study

| **Reference** | ***Wolbachia* isolate (host)** |
| --- | --- |
| GCF_013366805.1_ASM1336680v1_genomic | *Wolbachia* sp. 37PF *(Litomosoides brasiliensis*) |
| GCF_014771645.1_ASM1477164v1_genomic | *Candidatus Wolbachia massiliensis* PL13 |
| GCA_031260505.1_ASM3126050v1_genomic | *Wolbachia pipientis* Aly426_bin7 |
| GCF_030373985.1_ASM3037398v1_genomic | *Wolbachia pipientis w*Stur (*Syringophilopsis turdus*) |
| GCF_030295115.1_ASM3029511v1_genomic | *Wolbachia pipientis w*Hm-c |
| GCF_030295095.1_ASM3029509v1_genomic | *Wolbachia pipientis w*Hm-t |
| GCA_025781775.1_ASM2578177v1_genomic | *Wolbachia pipientis w*Dgra |
| GCF_024205405.1_ASM2420540v1_genomic | *Wolbachia pipientis* AH2012_wTtru (*Tetranychus truncatus*) |
| GCF_023661085.1_ASM2366108v1_genomic | *Wolbachia pipientis w*Oc (*Osmia caerulescens*) |
| GCF_022343925.1_ASM2234392v1_genomic | *Wolbachia pipientis* F3 |
| GCF_022343905.1_ASM2234390v1_genomic | *Wolbachia pipientis* F7_A |
| GCF_022343885.1_ASM2234388v1_genomic | *Wolbachia pipientis* F7_B |
| GCF_022343865.1_ASM2234386v1_genomic | *Wolbachia pipientis* donor |
| GCF_022343845.1_ASM2234384v1_genomic | *Wolbachia pipientis* F7_C |
| GCF_017604245.1_ASM1760424v1_genomic | *Wolbachia pipientis w*Cin2USA1 (*Rhagoletis cingulata*) |
| GCF_014129655.1_ASM1412965v1_genomic | *Wolbachia pipientis w*Ara (*Drosophila arawakana*) |
| GCF_014129615.1_ASM1412961v1_genomic | *Wolbachia pipientis w*Bor (*Drosophila borealis*) |
| GCF_014129565.1_ASM1412956v1_genomic | *Wolbachia pipientis w*Orie (*Drosophila orientacea*) |
| GCF_014129525.1_ASM1412952v1_genomic | *Wolbachia pipientis w*Tro (*Drosophila tropicalis*) |
| GCA_011090435.1_ASM1109043v1_genomic | *Wolbachia pipientis w*Cer2_DsimRC50 (*Drosophila simulans*) |
| GCF_007972745.1_ASM797274v1_genomic | *Wolbachia pipientis w*Mel_ZH26 (*Drosophila melanogaster*) |
| GCF_007972595.1_ASM797259v1_genomic | *Wolbachia pipientis w*Mel_I23 (*Drosophila melanogaster*) |
| GCF_007971685.1_ASM797168v1_genomic | *Wolbachia pipientis w*Mel_N25 (*Drosophila melanogaster*) |
| GCF_002318985.1_ASM231898v1_genomic | *Wolbachia pipientis* wAus (*Plutella australiana*) |
| GCF_001752665.1_ASM175266v1_genomic | *Wolbachia pipientis w*Ppe (*Pratylenchus penetrans*) |
| GCF_000742435.1_ASM74243v1_genomic | *Wolbachia pipientis w*Rec (*Drosophila recens*) |
| GCF_002204235.2_ASM220423v2_genomic | *Wolbachia* sp. *w*Wb (*Wuchereria bancrofti w*Wb) |
| GCF_000338395.1_ASM33839v1_genomic | *Wolbachia* sp. (*Wuchereria bancrofti*) |
| GCF_012030695.1_ASM1203069v1_genomic | *Wolbachia* sp. FR3 (*Brugia pahangi*) |
| This study (JBGGJS000000000) | *Wolbachia* sp. *w*Hhum (*Heterodera humuli)* |
| GCA_022836975.1_ASM2283697v1_genomic_wTex | *Wolbachia* sp. *w*Tex (*Helicotylenchus* sp.) |

**Supplementary Table 4** Sequencing yield and raw library metrics for six *Heterodera* species sequenced using the Illumina NovaSeq short read sequencing platform

|  | | ***H. australis*** | ***H. avenae*** | ***H. filipjevi*** | ***H. humuli*** | ***H. mani*** | ***H. trifolii*** |
| --- | --- | --- | --- | --- | --- | --- | --- |
| **General** | **fastp version** | 0.20.0 (https://github.com/OpenGene/fastp) | | | | | |
|  | **Sequencing** | 2×150 bp | 2×150 bp | 2×250 bp | 2×150 bp | 2×150 bp | 2×150 bp |
|  | **mean length before filtering** | 151bp, 151bp | 151bp, 151bp | 251bp, 251bp | 151bp, 151bp | 151bp, 151bp | 151bp, 151bp |
|  | **mean length after filtering** | 131bp, 131bp | 132bp, 132bp | 202bp, 203bp | 133bp, 133bp | 146bp, 146bp | 129bp, 129bp |
| **Before filtering** | **total reads (M)** | 196.7 | 80.0 | 19.0 | 122.5 | 52.5 | 65.6 |
|  | **total bases (Gb)** | 29.7 | 12.0 | 4.7 | 18.5 | 7.9 | 9.9 |
|  | **Q20 bases** | 95.76% | 93.70% | 90.30% | 94.14% | 93.74% | 94.87% |
|  | **Q30 bases** | 90% | 86.48% | 80.30% | 87.05% | 86.48% | 88.13% |
|  | **GC content** | 61.55% | 47.52% | 59.80% | 52.46% | 46.23% | 57.68% |
| **After filtering** | **total reads (M)** | 193.3 | 76.2 | 18.6 | 118.7 | 50.6 | 63.9 |
|  | **total bases (Gb)** | 25.4 | 10.09 | 3.7 | 15.8 | 7.4 | 8.2 |
|  | **Q20 bases** | 96.93% | 95.53% | 93.70% | 95.65% | 95.07% | 96.18% |
|  | **Q30 bases** | 91.73% | 88.78% | 84.60% | 89.03% | 88.14% | 89.95% |
|  | **GC content** | 61.34% | 45.50% | 58.80% | 51.78% | 45.48% | 57.24% |

M – Million

Gb – Gigabases

**Supplementary Table 5** The distribution of different phyla identified in the metagenomic samples of various *Heterodera* species. Each percentage represents the proportion of sequences assigned to the respective phylum in the metagenome of each species**.**

| ***Heterodera* species** | **Phyla present (%)** | | | | | | | | | | | |
| --- | --- | --- | --- | --- | --- | --- | --- | --- | --- | --- | --- | --- |
|  | **no-hit** | **Nematoda** | **Proteobacteria** | **Actinobacteria** | **Bacteriodetes** | **Ascomycota** | **Arthropoda** | **Firmicutes** | **Chordata** | **Planctomycetes** | **Others^1^** | **Undef^2^** |
| *H. australis* | 6.57 | 7.88 | 39.56 | 15.19 | 2.66 | 0.72 | 0.57 |  |  |  | 1.23 | 1.31 |
| *H. avenae* | 22.54 | 30.84 | 17.04 | 0.86 |  |  | 3.07 | 0.73 | 2 |  | 3.02 | 0.79 |
| *H. filipjevi* | 13.74 | 4.63 | 17.43 | 7.01 | 0.83 |  | 0.56 |  |  | 0.24 | 1.42 | 3.4 |
| *H. mani* | 18.97 | 35.87 | 22.24 | 0.62 |  | 0.58 | 2.1 |  | 1.97 |  | 2.12 | 1.11 |
| *H. humuli* | 11.74 | 25.53 | 23.33 | 3.51 | 2.74 | 2.1 |  |  | 2.64 |  | 6.2 | 1.5 |
| *H. trifolii* | 15.94 | 4.68 | 31.3 | 1.36 | 1.33 |  | 2.76 |  | 3.74 |  | 5.18 | 1.59 |

^1^The number of taxonomic groups plotted in the BlobPlot is ‘7’ and remaining groups are binned into the category ‘Others’

^2^Absence suppof a node at the taxonomic rank, bins the sequences into the ‘Undef’ category

**Supplementary Table 6** Average nucleotide identity matrix obtained for the percentage similarity comparison between different *Wolbachia* isolates obtained from NCBI GenBank and the Australian *w*Hhum isolate generated during this study (highlighted in yellow). The *w*Tex (*Helicotylenchus* sp.) isolate is highlighted in blue colour that is taken as the reference sequence here to extract *Wolbachia* reads from *H. humuli* data.

|  | GCF_000742435.1_ASM74243v1_genomic | GCA_011090435.1_ASM1109043v1_genomic | GCF_002204235.2_ASM220423v2_genomic | GCF_022343905.1_ASM2234390v1_genomic | GCF_023661085.1_ASM2366108v1_genomic | GCF_014129525.1_ASM1412952v1_genomic | GCF_013366805.1_ASM1336680v1_genomic | GCF_022343845.1_ASM2234384v1_genomic | GCF_001752665.1_ASM175266v1_genomic | GCF_030295095.1_ASM3029509v1_genomic | GCF_030373985.1_ASM3037398v1_genomic | GCF_024205405.1_ASM2420540v1_genomic | GCF_014129615.1_ASM1412961v1_genomic | GCF_014771645.1_ASM1477164v1_genomic | GCF_030295115.1_ASM3029511v1_genomic | GCF_007971685.1_ASM797168v1_genomic | GCF_014129565.1_ASM1412956v1_genomic | GCF_007972595.1_ASM797259v1_genomic | GCF_014129655.1_ASM1412965v1_genomic | GCA_031260505.1_ASM3126050v1_genomic | GCF_022343925.1_ASM2234392v1_genomic | GCF_022343885.1_ASM2234388v1_genomic | GCF_022343865.1_ASM2234386v1_genomic | GCA_025781775.1_ASM2578177v1_genomic | GCA_022836975.1_ASM2283697v1_genomic_wTex | GCF_017604245.1_ASM1760424v1_genomic | GCF_012030695.1_ASM1203069v1_genomic | GCF_007972745.1_ASM797274v1_genomic | wHhum_contigs | GCF_002318985.1_ASM231898v1_genomic |
| --- | --- | --- | --- | --- | --- | --- | --- | --- | --- | --- | --- | --- | --- | --- | --- | --- | --- | --- | --- | --- | --- | --- | --- | --- | --- | --- | --- | --- | --- | --- |
| GCF_000742435.1_ASM74243v1_genomic | 1 | 0.996226 | 0.856726 | 0.995657 | 0.859481 | 0.995522 | 0.852712 | 0.995688 | 0.832286 | 0.874634 | 0.86031 | 0.875286 | 0.994536 | 0.864811 | 0.874526 | 0.995655 | 0.965125 | 0.995625 | 0.995417 | 0.941712 | 0.995673 | 0.995692 | 0.995645 | 0.834375 | 0.851989986 | 0.996252 | 0.856811 | 0.995578 | 0.834535 | 0.875052 |
| GCA_011090435.1_ASM1109043v1_genomic | 0.996226 | 1 | 0.857215 | 0.995415 | 0.862569 | 0.995364 | 0.853133 | 0.995412 | 0.835478 | 0.878684 | 0.86116 | 0.877452 | 0.993483 | 0.866119 | 0.878747 | 0.995382 | 0.961563 | 0.995337 | 0.995669 | 0.941858 | 0.995402 | 0.995431 | 0.995353 | 0.834895 | 0.853976199 | 0.991 | 0.857206 | 0.995333 | 0.836077 | 0.876227 |
| GCF_002204235.2_ASM220423v2_genomic | 0.856726 | 0.857215 | 1 | 0.857213 | 0.859696 | 0.857348 | 0.906303 | 0.857213 | 0.841759 | 0.848378 | 0.859915 | 0.848298 | 0.857192 | 0.859431 | 0.848445 | 0.857218 | 0.857647 | 0.857216 | 0.857214 | 0.855986 | 0.857213 | 0.857213 | 0.857213 | 0.839146 | 0.869158879 | 0.85745 | 0.964605 | 0.857226 | 0.851109 | 0.848153 |
| GCF_022343905.1_ASM2234390v1_genomic | 0.995657 | 0.995415 | 0.857213 | 1 | 0.861008 | 0.994573 | 0.85307 | 0.999975 | 0.833507 | 0.877965 | 0.860854 | 0.877166 | 0.993723 | 0.865331 | 0.877463 | 0.999852 | 0.961361 | 0.999939 | 0.995258 | 0.940766 | 0.999939 | 0.999926 | 0.999864 | 0.834659 | 0.853918206 | 0.992563 | 0.857058 | 0.9998 | 0.835049 | 0.875105 |
| GCF_023661085.1_ASM2366108v1_genomic | 0.859481 | 0.862569 | 0.859696 | 0.861008 | 1 | 0.860411 | 0.855131 | 0.861149 | 0.842789 | 0.857997 | 0.867998 | 0.853791 | 0.86111 | 0.862878 | 0.857151 | 0.8613 | 0.862214 | 0.861151 | 0.860428 | 0.858704 | 0.861149 | 0.861149 | 0.861146 | 0.838738 | 0.857401448 | 0.863782 | 0.859488 | 0.861302 | 0.862747 | 0.854853 |
| GCF_014129525.1_ASM1412952v1_genomic | 0.995522 | 0.995364 | 0.857348 | 0.994573 | 0.860411 | 1 | 0.853028 | 0.994553 | 0.83363 | 0.877462 | 0.860815 | 0.877459 | 0.994362 | 0.86533 | 0.87727 | 0.994522 | 0.962916 | 0.994474 | 0.996391 | 0.941337 | 0.994539 | 0.994551 | 0.994507 | 0.833875 | 0.853925124 | 0.992833 | 0.856984 | 0.994493 | 0.836661 | 0.87527 |
| GCF_013366805.1_ASM1336680v1_genomic | 0.852712 | 0.853133 | 0.906303 | 0.85307 | 0.855131 | 0.853028 | 1 | 0.853197 | 0.84651 | 0.844802 | 0.85423 | 0.844269 | 0.852693 | 0.854278 | 0.844844 | 0.853193 | 0.852968 | 0.853195 | 0.853023 | 0.85233 | 0.853197 | 0.853197 | 0.853195 | 0.838177 | 0.867413035 | 0.853296 | 0.905479 | 0.853193 | 0.863352 | 0.844605 |
| GCF_022343845.1_ASM2234384v1_genomic | 0.995688 | 0.995412 | 0.857213 | 0.999975 | 0.861149 | 0.994553 | 0.853197 | 1 | 0.833507 | 0.877956 | 0.860854 | 0.877157 | 0.993736 | 0.865331 | 0.877454 | 0.999892 | 0.961361 | 0.999979 | 0.995287 | 0.940766 | 0.999982 | 0.999964 | 0.999905 | 0.834659 | 0.853918206 | 0.992583 | 0.857058 | 0.999842 | 0.835049 | 0.875095 |
| GCF_001752665.1_ASM175266v1_genomic | 0.832286 | 0.835478 | 0.841759 | 0.833507 | 0.842789 | 0.83363 | 0.84651 | 0.833507 | 1 | 0.845579 | 0.853062 | 0.845857 | 0.834763 | 0.840188 | 0.845543 | 0.832934 | 0.833515 | 0.832934 | 0.833062 | 0.836742 | 0.832934 | 0.832934 | 0.832934 | 0.842119 | 0.834007734 | 0.833282 | 0.849312 | 0.832906 | 0.836883 | 0.845901 |
| GCF_030295095.1_ASM3029509v1_genomic | 0.874634 | 0.878684 | 0.848378 | 0.877965 | 0.857997 | 0.877462 | 0.844802 | 0.877956 | 0.845579 | 1 | 0.850899 | 0.981734 | 0.876175 | 0.857403 | 0.999098 | 0.877724 | 0.879335 | 0.87774 | 0.876498 | 0.874951 | 0.877769 | 0.877775 | 0.877762 | 0.837832 | 0.841893628 | 0.882452 | 0.847978 | 0.877717 | 0.840392 | 0.986332 |
| GCF_030373985.1_ASM3037398v1_genomic | 0.86031 | 0.86116 | 0.859915 | 0.860854 | 0.867998 | 0.860815 | 0.85423 | 0.860854 | 0.853062 | 0.850899 | 1 | 0.851012 | 0.860633 | 0.862672 | 0.851258 | 0.860932 | 0.860186 | 0.86093 | 0.861264 | 0.859048 | 0.860929 | 0.860929 | 0.860926 | 0.838099 | 0.876894224 | 0.861213 | 0.859322 | 0.860932 | 0.850669 | 0.85153 |
| GCF_024205405.1_ASM2420540v1_genomic | 0.875286 | 0.877452 | 0.848298 | 0.877166 | 0.853791 | 0.877459 | 0.844269 | 0.877157 | 0.845857 | 0.981734 | 0.851012 | 1 | 0.875275 | 0.853443 | 0.981323 | 0.876791 | 0.878467 | 0.876708 | 0.876319 | 0.874683 | 0.87677 | 0.876778 | 0.876784 | 0.838143 | 0.836048345 | 0.877873 | 0.848734 | 0.876779 | 0.847831 | 0.981065 |
| GCF_014129615.1_ASM1412961v1_genomic | 0.994536 | 0.993483 | 0.857192 | 0.993723 | 0.86111 | 0.994362 | 0.852693 | 0.993736 | 0.834763 | 0.876175 | 0.860633 | 0.875275 | 1 | 0.865384 | 0.876065 | 0.99366 | 0.963011 | 0.993539 | 0.994337 | 0.941418 | 0.993691 | 0.993688 | 0.993654 | 0.834905 | 0.858607141 | 0.992735 | 0.856962 | 0.993637 | 0.838323 | 0.87493 |
| GCF_014771645.1_ASM1477164v1_genomic | 0.864811 | 0.866119 | 0.859431 | 0.865331 | 0.862878 | 0.86533 | 0.854278 | 0.865331 | 0.840188 | 0.857403 | 0.862672 | 0.853443 | 0.865384 | 1 | 0.857228 | 0.865568 | 0.86706 | 0.865597 | 0.865464 | 0.863033 | 0.865562 | 0.865562 | 0.865561 | 0.836886 | 0.847374901 | 0.871881 | 0.859402 | 0.865567 | 0.842154 | 0.853137 |
| GCF_030295115.1_ASM3029511v1_genomic | 0.874526 | 0.878747 | 0.848445 | 0.877463 | 0.857151 | 0.87727 | 0.844844 | 0.877454 | 0.845543 | 0.999098 | 0.851258 | 0.981323 | 0.876065 | 0.857228 | 1 | 0.877086 | 0.878322 | 0.877038 | 0.875676 | 0.874792 | 0.87706 | 0.877067 | 0.877074 | 0.837861 | 0.841080429 | 0.882466 | 0.848084 | 0.877079 | 0.840342 | 0.986027 |
| GCF_007971685.1_ASM797168v1_genomic | 0.995655 | 0.995382 | 0.857218 | 0.999852 | 0.8613 | 0.994522 | 0.853193 | 0.999892 | 0.832934 | 0.877724 | 0.860932 | 0.876791 | 0.99366 | 0.865568 | 0.877086 | 1 | 0.961331 | 0.999953 | 0.995238 | 0.940838 | 0.999882 | 0.999867 | 0.999939 | 0.834664 | 0.853918206 | 0.992564 | 0.857058 | 0.999936 | 0.835049 | 0.875121 |
| GCF_014129565.1_ASM1412956v1_genomic | 0.965125 | 0.961563 | 0.857647 | 0.961361 | 0.862214 | 0.962916 | 0.852968 | 0.961361 | 0.833515 | 0.879335 | 0.860186 | 0.878467 | 0.963011 | 0.86706 | 0.878322 | 0.961331 | 1 | 0.96184 | 0.963124 | 0.941054 | 0.961891 | 0.961891 | 0.96185 | 0.835234 | 0.844833719 | 0.960586 | 0.856858 | 0.961856 | 0.840542 | 0.875569 |
| GCF_007972595.1_ASM797259v1_genomic | 0.995625 | 0.995337 | 0.857216 | 0.999939 | 0.861151 | 0.994474 | 0.853195 | 0.999979 | 0.832934 | 0.87774 | 0.86093 | 0.876708 | 0.993539 | 0.865597 | 0.877038 | 0.999953 | 0.96184 | 1 | 0.995211 | 0.940731 | 0.999969 | 0.999955 | 0.999957 | 0.834659 | 0.853918206 | 0.992517 | 0.857061 | 0.99992 | 0.835049 | 0.875031 |
| GCF_014129655.1_ASM1412965v1_genomic | 0.995417 | 0.995669 | 0.857214 | 0.995258 | 0.860428 | 0.996391 | 0.853023 | 0.995287 | 0.833062 | 0.876498 | 0.861264 | 0.876319 | 0.994337 | 0.865464 | 0.875676 | 0.995238 | 0.963124 | 0.995211 | 1 | 0.941583 | 0.995899 | 0.995925 | 0.995861 | 0.834755 | 0.8560225 | 0.994788 | 0.857032 | 0.995771 | 0.83796 | 0.875576 |
| GCA_031260505.1_ASM3126050v1_genomic | 0.941712 | 0.941858 | 0.855986 | 0.940766 | 0.858704 | 0.941337 | 0.85233 | 0.940766 | 0.836742 | 0.874951 | 0.859048 | 0.874683 | 0.941418 | 0.863033 | 0.874792 | 0.940838 | 0.941054 | 0.940731 | 0.941583 | 1 | 0.940958 | 0.940958 | 0.940956 | 0.83793 | 0.851024778 | 0.941061 | 0.856328 | 0.941032 | 0.843326 | 0.874853 |
| GCF_022343925.1_ASM2234392v1_genomic | 0.995673 | 0.995402 | 0.857213 | 0.999939 | 0.861149 | 0.994539 | 0.853197 | 0.999982 | 0.832934 | 0.877769 | 0.860929 | 0.87677 | 0.993691 | 0.865562 | 0.87706 | 0.999882 | 0.961891 | 0.999969 | 0.995899 | 0.940958 | 1 | 0.999954 | 0.999897 | 0.834659 | 0.853918206 | 0.992563 | 0.857058 | 0.999832 | 0.835049 | 0.875095 |
| GCF_022343885.1_ASM2234388v1_genomic | 0.995692 | 0.995431 | 0.857213 | 0.999926 | 0.861149 | 0.994551 | 0.853197 | 0.999964 | 0.832934 | 0.877775 | 0.860929 | 0.876778 | 0.993688 | 0.865562 | 0.877067 | 0.999867 | 0.961891 | 0.999955 | 0.995925 | 0.940958 | 0.999954 | 1 | 0.999878 | 0.834659 | 0.853918206 | 0.992601 | 0.857058 | 0.999817 | 0.835049 | 0.875103 |
| GCF_022343865.1_ASM2234386v1_genomic | 0.995645 | 0.995353 | 0.857213 | 0.999864 | 0.861146 | 0.994507 | 0.853195 | 0.999905 | 0.832934 | 0.877762 | 0.860926 | 0.876784 | 0.993654 | 0.865561 | 0.877074 | 0.999939 | 0.96185 | 0.999957 | 0.995861 | 0.940956 | 0.999897 | 0.999878 | 1 | 0.834659 | 0.853918206 | 0.992517 | 0.857055 | 0.999889 | 0.835049 | 0.875111 |
| GCA_025781775.1_ASM2578177v1_genomic | 0.834375 | 0.834895 | 0.839146 | 0.834659 | 0.838738 | 0.833875 | 0.838177 | 0.834659 | 0.842119 | 0.837832 | 0.838099 | 0.838143 | 0.834905 | 0.836886 | 0.837861 | 0.834664 | 0.835234 | 0.834659 | 0.834755 | 0.83793 | 0.834659 | 0.834659 | 0.834659 | 1 | 0.836319295 | 0.834926 | 0.837699 | 0.834823 | 0.864793 | 0.837942 |
| GCA_022836975.1_ASM2283697v1_genomic_wTex | 0.85199 | 0.853976 | 0.869159 | 0.853918 | 0.857401 | 0.853925 | 0.867413 | 0.853918 | 0.834008 | 0.841894 | 0.876894 | 0.836048 | 0.858607 | 0.847375 | 0.84108 | 0.853918 | 0.844834 | 0.853918 | 0.856022 | 0.851025 | 0.853918 | 0.853918 | 0.853918 | 0.836319 | 1 | 0.85393 | 0.860521 | 0.853896 | 0.924461 | 0.842488 |
| GCF_017604245.1_ASM1760424v1_genomic | 0.996252 | 0.991 | 0.85745 | 0.992563 | 0.863782 | 0.992833 | 0.853296 | 0.992583 | 0.833282 | 0.882452 | 0.861213 | 0.877873 | 0.992735 | 0.871881 | 0.882466 | 0.992564 | 0.960586 | 0.992517 | 0.994788 | 0.941061 | 0.992563 | 0.992601 | 0.992517 | 0.834926 | 0.853930287 | 1 | 0.857459 | 0.992593 | 0.836043 | 0.8764 |
| GCF_012030695.1_ASM1203069v1_genomic | 0.856811 | 0.857206 | 0.964605 | 0.857058 | 0.859488 | 0.856984 | 0.905479 | 0.857058 | 0.849312 | 0.847978 | 0.859322 | 0.848734 | 0.856962 | 0.859402 | 0.848084 | 0.857058 | 0.856858 | 0.857061 | 0.857032 | 0.856328 | 0.857058 | 0.857058 | 0.857055 | 0.837699 | 0.860520592 | 0.857459 | 1 | 0.857119 | 0.847536 | 0.848154 |
| GCF_007972745.1_ASM797274v1_genomic | 0.995578 | 0.995333 | 0.857226 | 0.9998 | 0.861302 | 0.994493 | 0.853193 | 0.999842 | 0.832906 | 0.877717 | 0.860932 | 0.876779 | 0.993637 | 0.865567 | 0.877079 | 0.999936 | 0.961856 | 0.99992 | 0.995771 | 0.941032 | 0.999832 | 0.999817 | 0.999889 | 0.834823 | 0.853895771 | 0.992593 | 0.857119 | 1 | 0.835049 | 0.875092 |
| wHhum_contigs (This Study) | 0.834535 | 0.836077 | 0.851109 | 0.835049 | 0.862747 | 0.836661 | 0.863352 | 0.835049 | 0.836883 | 0.840392 | 0.850669 | 0.847831 | 0.838323 | 0.842154 | 0.840342 | 0.835049 | 0.840542 | 0.835049 | 0.83796 | 0.843326 | 0.835049 | 0.835049 | 0.835049 | 0.864793 | 0.924460703 | 0.836043 | 0.847536 | 0.835049 | 1 | 0.851451 |
| GCF_002318985.1_ASM231898v1_genomic | 0.875052 | 0.876227 | 0.848153 | 0.875105 | 0.854853 | 0.87527 | 0.844605 | 0.875095 | 0.845901 | 0.986332 | 0.85153 | 0.981065 | 0.87493 | 0.853137 | 0.986027 | 0.875121 | 0.875569 | 0.875031 | 0.875576 | 0.874853 | 0.875095 | 0.875103 | 0.875111 | 0.837942 | 0.842487884 | 0.8764 | 0.848154 | 0.875092 | 0.851451 | 1 |
